# Mining the entire Protein DataBank for frequent spatially cohesive amino acid patterns

**DOI:** 10.1186/s13040-015-0038-4

**Published:** 2015-01-31

**Authors:** Pieter Meysman, Cheng Zhou, Boris Cule, Bart Goethals, Kris Laukens

**Affiliations:** 1 Advanced Database Research and Modelling (ADReM), Department of Mathematics and Computer Science, University of Antwerp, Antwerp, Belgium; 2 Biomedical Informatics Research Center Antwerp (biomina), University of Antwerp/Antwerp University Hospital, Edegem, Belgium

**Keywords:** Protein structure, Frequent pattern mining, Thermostability, Protein-DNA complexes

## Abstract

**Background:**

The three-dimensional structure of a protein is an essential aspect of its functionality. Despite the large diversity in protein structures and functionality, it is known that there are common patterns and preferences in the contacts between amino acid residues, or between residues and other biomolecules, such as DNA. The discovery and characterization of these patterns is an important research topic within structural biology as it can give fundamental insight into protein structures and can aid in the prediction of unknown structures.

**Results:**

Here we apply an efficient spatial pattern miner to search for sets of amino acids that occur frequently in close spatial proximity in the protein structures of the Protein DataBank. This allowed us to mine for a new class of amino acid patterns, that we term FreSCOs (Frequent Spatially Cohesive Component sets), which feature synergetic combinations. To demonstrate the relevance of these FreSCOs, they were compared in relation to the thermostability of the protein structure and the interaction preferences of DNA-protein complexes. In both cases, the results matched well with prior investigations using more complex methods on smaller data sets.

**Conclusions:**

The currently characterized protein structures feature a diverse set of frequent amino acid patterns that can be related to the stability of the protein molecular structure and that are independent from protein function or specific conserved domains.

**Electronic supplementary material:**

The online version of this article (doi:10.1186/s13040-015-0038-4) contains supplementary material, which is available to authorized users.

## Background

Proteins are primarily composed of a long chain of amino acids that is folded into a complex three-dimensional structure. This spatial structure of a protein is an essential component in its functionality and is thus subjected to evolutionary pressures to optimize the inter-residue contacts that support it. For example, the proteins of thermophilic organisms are known to contain specific adaptations in their amino acid configurations and content to stabilize the molecular protein structure in the high temperature living environment in which these species live [1,2]. Despite the large diversity in protein structures and functionality, it is known that there are common patterns and contact preferences between residues, or even between residues and other biomolecules, such as DNA.

Different methods have been described to explore the amino acid content of a protein and their interactions for a variety of goals. Within the scope of molecular modelling, frequent representations of common inter-residue contacts involve protein contact maps (PCM) or adjacency matrices [3,4]. These types of representations transform the three-dimensional organisation of protein residues into a two-dimensional interaction map. These maps can then be mined to identify common patterns of residues in contact that occur in multiple different protein structures [5]. A variety of techniques have been applied to this problem, such as support vector machines [6], hidden markov models [7,8] and neural networks [9]. These patterns are then commonly used for protein fold prediction, often by first predicting the PCM from the amino acid sequence [10,11]. A second type of mining applied to protein structures involves the discovery of specific re-occurring local structures or amino acid motifs that can be related to a given protein function or family [12]. A common approach to finding such patterns involves the transformation of the protein structure into a residue graph where edges represent interactions or close proximity. Common patterns can then be identified through subgraph mining techniques, which can then be used for the functional characterization of other protein structures [13-16].

The RSCB Protein DataBank (PDB) is the primary collection of protein molecular structures [17]. It contains the full atomic molecular structures for more than 90 000 proteins and protein complexes at the time of writing. This represents the largest collection of protein molecular structures that is nowadays available and has been the subject of many data mining initiatives. However most data mining approaches are only applied to a small subset of the PDB database, typically no more than a few hundred structures. The main hurdle with scaling these approaches up to larger data sets, is that the computation time and memory usage scale up too, in most cases in a more than linear fashion due to the complexity of three-dimensional molecular structures data.

Frequent pattern mining is a data mining technique that was developed to identify elements that often co-occur within a data set. The archetypical usage case is the ‘market basket’ problem, where the goal is to identify which items are often bought together based on the transactions made at a supermarket. Despite the simplicity of this problem, creating an algorithm that can solve it in an accurate and rapid manner is not trivial. A significant amount of research has therefore gone into solving this problem as efficiently as possible and many algorithms now exist that address it. Many common biological challenges can be readily translated into the ‘market basket’ problem, and therefore frequent pattern mining has seen significant use in bioinformatics applications [18]. Relevant to the field of structural biology, frequent pattern mining has been applied to discover the common patterns that are present in PCMs [8] and for secondary structure prediction [19]. A recent extension of the frequent pattern mining field is the concept of pattern *cohesion*. This metric scores an item set based on the physical distance of the typical items within the set; i.e. items that are far apart can be said to have a low cohesion, while items that are proximal have a high cohesion [20]. While originally proposed for sequence data, this definition of cohesion can be readily extended to three dimensions. This has recently been implemented in a frequent item set miner that can rapidly find patterns of amino acids that often co-occur within a set of protein structures, without the need to convert the protein structure into a contact map or a graph [21]. We use the term FreSCOs (Frequent Spatially Cohesive Component sets) to refer to this type of pattern class.

We have shown that the patterns retrieved on such a protein data set mostly involve combinations of three or four amino acids that frequently occur in close proximity [21]. Thus these FreSCOs are more complex than the pairwise interaction between two residues, but not so extensive that they are unique to a specific protein or protein family. In this paper, we investigate what amino acid patterns are common in a large collection of protein structures and compare the discovered patterns to those that have been described previously using other classes of methods on smaller data sets. Further we explore the possibility to find FreSCOs that can be linked to the optimal growth temperature of the organism or to preferred protein-DNA interactions.

## Methods

### Data set

Two large collections of three-dimensional structures are used in this study. The first is the collection of all structures contained within the RCSB PDB database, obtained on the 3rd of May, 2013 [17]. Only non-redundant protein sequences were retained as annotated by the Vector Alignment Search Tool in the non-redundant PDB chain set at different sequence similarity cut-offs [22]. The largest set of proteins that was considered in this manner, where structures with a BLAST p-value lower than 10^−80^ were considered redundant, contains 32 142 protein molecules from a large variety of organisms. As using more stringent redundancy cut-offs had little effect on the resulting patterns [see Additional file 1], we chose to use the largest set for increased statistical power. The second three-dimensional structure data set is a collection of 2 901 DNA-protein complex molecular structures (less than 90% protein sequence identity) as obtained from the RCSB PDB database on the 15th of October, 2013. The data set presented to the pattern mining algorithm used the C_α_ atom for protein residues and the N_1_ atom for DNA bases to determine the coordinates for the given label, i.e. the type of amino acid or base.

### Pattern mining algorithm

The algorithm used in this paper is based on the principles of frequent pattern mining and the data mining concept of *cohesion*. In brief, the algorithm considers all possible amino acid combinations and identifies those combinations that are both frequent in the set of protein structures and that consist of amino acids in close average proximity. Hereafter these patterns of frequent spatially cohesive amino acids will be referred to as ‘FreSCOs’. This method can be sped up by pruning the possible search space with little to no loss in accuracy [23]. The next section introduces the definitions and algorithmic framework behind the pattern miner.

The three-dimensional structure of a protein can be considered as a list of points where each point *v* is a pair (*a*,*c*) consisting of an item *a* ∈ *I*, where *I* is the set of all possible amino acids and a coordinate *c* ∈ **R**^3^. We can then represent a protein as a data object *d* = {*v*_*1*_, …, *v*_*l*_}, where *l* is the number of amino acids in the protein sequence. The set of all proteins within a given data set is denoted by *D*. We assume that two points can never occur at the same position, i.e. with the same coordinate. On the other hand, an amino acid *a*_*i*_ may occur many times at different positions in a protein *d*_*g*_ with *V*_*gi*_ denoting the set of these points*.*

A subset *X* = {*a*_*1*_, …, *a*_*k*_} ⊆ *I* is termed a pattern of length *k*. Within the scope of this paper, we are interested in patterns with both a high *support*, i.e. those that occur often, and with a high *cohesion*, i.e. where the items are, on average, in close spatial proximity. For a given pattern *X*, we denote the set of all data objects that contain all items of *X* as *N(X)* = {*d* ∈ *D*|∀*a* ∈ *X*, ∃(*a*, *c*) ∈ *d*}. The *support* of *X* in a data set *D* is then defined as

$$ S(X)=\frac{\left|N\right.\left.(X)\right|}{\left|D\right|} $$

The concept of *cohesion* within the scope of data mining can be readily extended to three-dimensional space. Given a set of points *V* = {*v*_*1*_, …, *v*_*k*_}, let *MB(V)* denote the ball with the smallest radius that contains *V*, i.e. the *smallest enclosing ball*. It has been shown that *MB(V)* always exists and is unique [24]. Intuitively, we consider the points *V* to be in close spatial proximity if the radius of *MB(V)* is small enough. Given a pattern *X* = {*a*_*1*_, …, *a*_*k*_}, assume that each amino acid *a*_*i*_ occurs n_i_ times in a protein *d*_*g*_ ∈ *N(X)*. Within a single protein *d*_*g*_ that contains X, *R*_*g*_ is then defined as the radius of the smallest enclosing ball for a combination of *V* = {*v*_*1*_, …, *v*_*k*_} that matches *X* = {*a*_*1*_, …, *a*_*k*_}. We then define the *cohesive radius* of *X* in *D* as

$$ R(X)=\frac{{\displaystyle \sum_{d_e\in N(X)}{R}_g(X)}}{\left|N(X)\right|} $$

The pattern miner theoretically enumerates every possible amino acid combination for their support and cohesion radius value, and will return those patterns that exceed the given cut-off for both. The utilized Apriori-like algorithm [25] speeds up this pattern search by using several properties of the support and cohesion metrics [23]. As the most efficient version of this algorithm is used for this study, there is no longer a necessity of explicitly including secondary structure information in the miner. In this manner the data set is not limited by any additional annotation being available and allows inclusions of a much larger set of protein structures. The mining procedure is able to return results after about 6 000 s (1 h40) when run on a local server (one 2.67 Ghz core, 24 Gb RAM) for the largest non-redundant PDB data set (almost 25 Gb of PDB files).

### Significance of the discovered patterns

The pattern miner reveals all FreSCOs that are both frequent and in close proximity. Intuitively, one realizes that patterns matching very common amino acids will have on average a smaller enclosing ball by chance than those that are less common. To compensate for this phenomenon, a background distribution is computed for each described pattern based on a permuted set of protein structures. In this background set, the amino acid labels have been randomized for each protein while keeping the overall structure (i.e. the coordinates) identical. The pattern miner is then applied to this permuted set to compute a new cohesion value. Due to the nature of the permutation, the frequency of the amino acids and the patterns will remain the same, as the protein amino acid content does not change. This permutation is repeated ten times and the resulting cohesion values are used to estimate a background distribution for the cohesion value of each pattern. As these background scores seem to follow a normal distribution [see Additional file 2 for an example], the probability that the true cohesion value follows the same background distribution can be computed. The p-value cut-off used is 0.01 with a Bonferroni correction for multiple testing.

### Domain enrichment

To identify the known conserved domains present in the studied protein set, the sequence of the non-redundant protein structures extracted from PDB were run through the Pfam sequence search web tool [26]. Domains were identified in 28 665 protein sequences, with a total of 58 459 domain-to-protein assignments. These assignments were compared to the FreSCOs by matching the sequence position of the residues that make up the smallest enclosing ball and the residues that define the conserved domains. In other words, for each FreSCO it was evaluated if the residues that match it are more or less likely to occur in any conserved protein domains. The significance of the overlap between the pattern residues and the domain residues was evaluated using a hypergeometric distribution at a P-value of 0.01 with a Bonferonni correction for multiple testing.

### Gene ontology enrichment

The gene ontology assignments to each protein chain that exist within the PDB database can be used to assess the overlap between specific protein functional characteristics and FreSCOs. To this end, each GO term associated that is annotated to at least 10 protein structures in the non-redundant PDB data set was checked for enrichment of FreSCOs. Gene ontology terms from the molecular function, cellular component and biological process trees were considered. However as most FreSCOs have a frequency of more than 90%, only the most cohesive matches within a protein structure were regarded as a match in this analysis. To this end, only matches where the residues occurred within a minimal enclosing ball with a radius less than 3 Å were included. The P-value for enrichment was calculated based on a hypergeometric distribution against specific FreSCOs occurring in a set of protein structures with a given gene ontology by chance.

### Optimal growth temperature

The optimal growth temperature (OGT) for different prokaryotic species was retrieved from the BacDive database on the 17th of December, 2013 [27]. Organisms with an OGT of 37°C were filtered out as these mainly concern pathogenic species whose protein content might bias the analysis [28,29]. Using the species annotation available through PDB, each molecular structure was linked to the species of origin. In this manner 4 952 protein structures from the non-redundant data set could be matched with an OGT. The FreSCOs extracted from the PDB data set can then be related to the OGT by calculating the Spearman correlation between the cohesive radius of the pattern match in a protein structure and the corresponding OGT. As a lower cohesive radius implies a tighter pattern, positive correlation is an indication for patterns with a higher relevance in lower temperatures, and negative correlation for higher temperatures. The p-value cut-off used is 0.01 with a Bonferroni correction for multiple testing.

## Results and discussion

### Triplet patterns feature synergetic combinations

**Figure 1:**
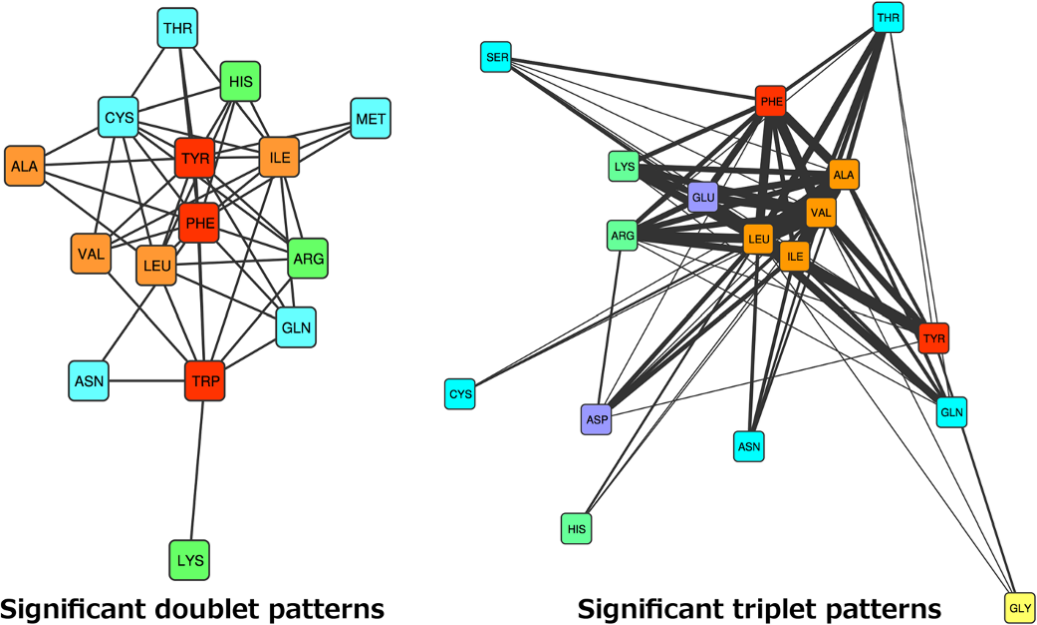
**Graph overview of the significant FreSCOs found in the non-redundant PDB data set.** Doublet (left) and triplet (right) patterns extracted from the non-redundant protein structure data set that had a cohesive radius significantly less than expected at random. Each node in the graph represents an amino acid and each edge represents a pattern that includes the two connecting amino acids where the edge width is scaled to the number of patterns. The nodes are colored based on the properties of the amino acid: aliphatic (orange), aromatic (red), polar (blue), acidic (purple), basic (green) and glycine (yellow).

The entire non-redundant PDB data set can be mined for FreSCOs, i.e. patterns of amino acids that frequently occur in close proximity. Amino acids in close proximity are defined by a maximum cohesive radius of 4.5 Å for the purposes of this analysis, which corresponds to the distance between the C_α_ atoms of two residues that are typically considered as interacting [9]. The support of the patterns is set to 0.60, much lower than the frequency of the individual amino acids [see Additional file 1] and further reduction of this parameter does not reveal any additional patterns. In the set of 32 142 non-redundant protein structures, 185 and 260 combinations of respectively two (doublet) and three (triplet) amino acids matched these criteria. However, one immediate observation is that the most cohesive FreSCOs are those composed of the most frequent amino acids. This makes sense because frequent amino acids will occur closer together even if it is simply by chance. We can correct for this bias by comparing these results to the cohesive radius of randomized proteins with similar amino acid frequencies. When we removed those FreSCOs whose cohesive radius did not significantly differ from random, 48 doublet and 104 triplet patterns remained [see Additional file 3]. These FreSCOs therefore represent amino acids that are significantly more often in close proximity than one would expect. Given the underlying relationship where triplet patterns can be considered built up out of two doublet patterns, one would expect similar patterns to emerge; however this is clearly not the case. As can be seen in Figure 1, the significant triplet patterns differ greatly from the doublet patterns. For example, the triplet patterns display several strong links between acidic (ASP, GLU) and basic (ARG, LYS) amino acids. These patterns are not present in the doublet list, yet are highly relevant, as previous studies have found that interactions between these residues form the bulk of the ionic bonds in protein structures [30,31]. In addition, there is a tight clustering of the aliphatic amino acids in the triplet patterns, as can be expected given that these amino acids typically form the hydrophobic protein core [32,33]. This is in contrast to the doublet patterns, where the aliphatic amino acids do cluster together but have few connections. We can postulate several reasons for the difference between the doublet and triplet patterns. Due to the correction step, many triplet patterns featuring only frequent amino acids might have been filtered out. However the triplet patterns still retain several combinations of frequent amino acids, such as all possible combinations with the four aliphatic amino acids: ALA-VAL-LEU, ALA-LEU-ILE, ALA-VAL-ILE and VAL-LEU-ILE. Another possible reason is the difference between the doublet and triplet patterns in terms of information content with regards to the positioning and the functionality of the pattern. A combination of two aliphatic amino acids may easily occur simply by chance, while an aliphatic triplet is more indicative of a hydrophobic core. It may be that the chemical interaction is only present between two of the amino acids, such as the FreSCOs featuring a basic and an acidic amino acid, and the third amino acid provides the necessary context for this interaction, for example a hydrophilic or hydrophobic residue. In general, these triplet patterns thus represent a synergetic effect between the three possible interaction pairs in the protein structures. Given their increased information content and their intrinsic novelty compared to patterns found with contact map approaches, the triplet (or larger) patterns will be the main focus of the next analyses.

### The discovered patterns seem to represent common building blocks

**Figure 2:**
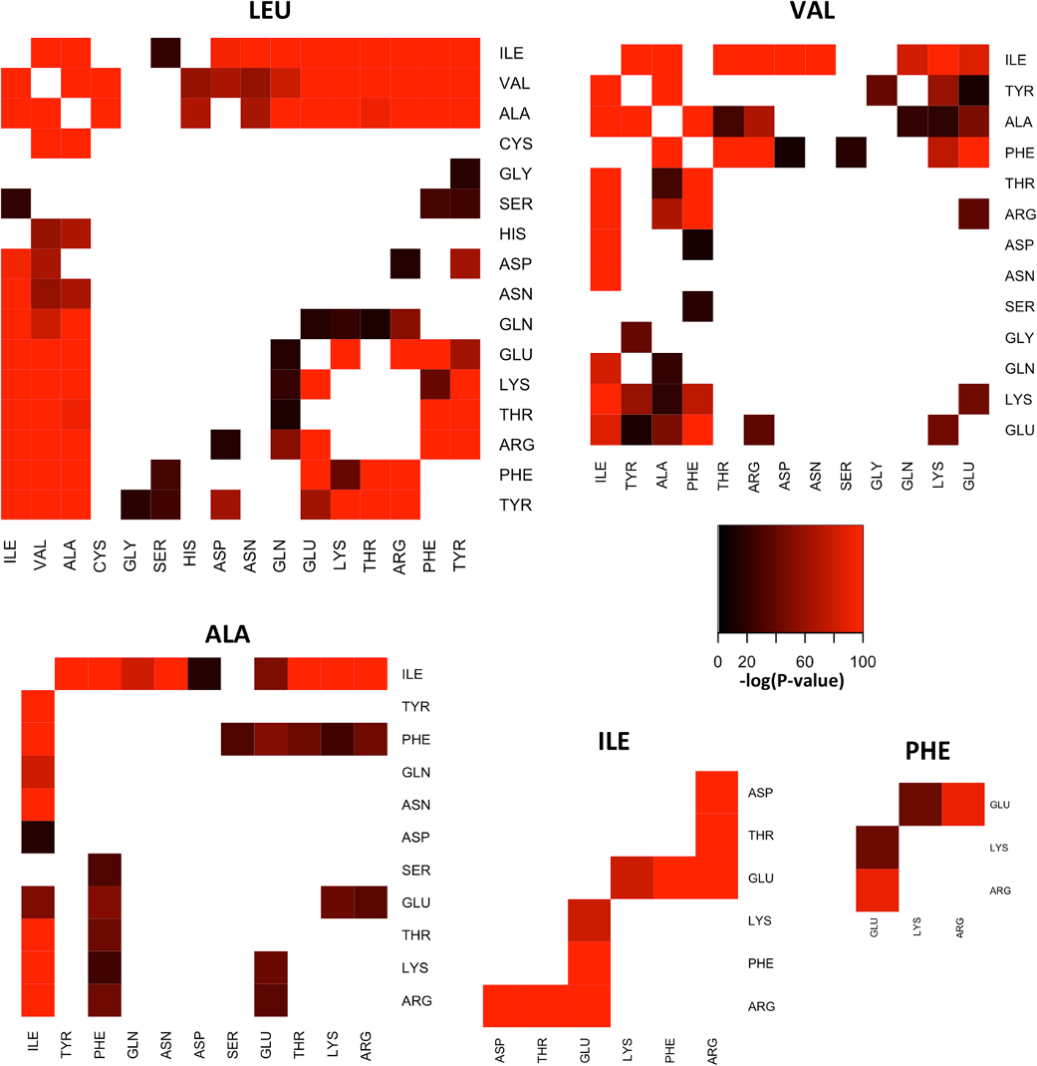
**Significant triplet FreSCOs extracted from the non-redundant PDB data set.** Plotted in the heatmaps is the logarithm of the p-value of a randomized protein data set having an average cohesion radius that is lower or the same for each pattern. The heatmaps were plotted so that every found pattern occurs exactly once using the smallest amount of heatmaps.

**Figure 3:**
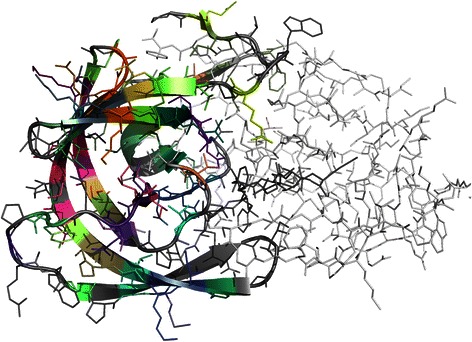
**Illustration of the pattern distribution in a mined protein structure.** Protein structure of the HIV-1 protease homodimer [PDB:1DAZ] [36]. Only one of the homodimer chains was mined for FreSCOs (represented in cartoon and stick format), the other is represented in light grey (stick format only). Significant triplet FreSCOs in the mined protein structure are visualized by colorization of the best matching residues, i.e. those with the smallest enclosing ball within this protein structure. Each pattern is assigned a unique color based on amino acid content. Residues that do not match any significant patterns are colored in dark grey.

As can be seen in Figure 2, all triplet FreSCOs contain at least one of the four aliphatic residues, except for the patterns PHE-GLU-ARG and PHE-GLU-LYS. Note that these patterns are not limited to any specific type of protein, in contrast to those documented for more complex spatial residue motifs [13-15]. Most FreSCOs described here have a support of more than 0.9, thus they occur in more than 90% of all the analyzed proteins. Given their ubiquitous presence, they are therefore more akin to common building blocks of the protein structure. In addition, the FreSCOs are unrelated to larger types of protein building blocks, such as the characterized conserved protein domains, as FreSCOs appear in a larger protein set and are not necessarily limited to the conserved regions. To demonstrate this fact, the residues that form these patterns can be matched with the conserved domains present in these proteins. Screening the protein sequences with the Pfam models [26] reveals that 77.18% of the residues in our protein set form part of a characterized protein domain. While a large fraction of the FreSCOs are significantly associated with conserved domains, seven patterns were found to avoid occurring in any protein domains [see Additional file 4]. These depleted patterns thus tend to occur outside of the known conserved regions of the protein structures and feature mostly combinations of glutamate, lysine, glutamine, leucine and isoleucine. However our analysis also showed that the amino acids themselves are not uniformly distributed across conserved domains and non-conserved regions. For example, the conserved domains themselves are highly enriched for cysteine but are highly depleted for proline and serine [see Additional file 5]. These amino acid preferences can partly explain the enrichment or depletion of specific FreSCOs in the conserved domains, as conserved domains seem to be significantly depleted for glutamate, glutamine and lysine residues. The residues that match the discovered FreSCOs thus do not all cluster at specific functional regions or a certain type of secondary structure, as shown for an example protein structure in Figure 3. A gene ontology analysis reveals that certain gene ontology terms are highly enriched for specific FreSCOs with a clear functional role [see Additional file 6]. For example, protein structures annotated as membrane proteins are highly enriched for FreSCOs consisting of mainly hydrophobic amino acids, such as PHE-VAL-LEU. The majority of significant FreSCO associations arises from the molecular function ontology tree. A large amount of FreSCOs are enriched in proteins that bind nucleotides, or have a transferase or oxidoreductase activity. An exhaustive review of all enriched FreSCOs in these gene ontology terms would exceed the scope of this paper. However for the nucleotide-binding proteins many FreSCOs include arginine, which is known to mediate a large number of nucleotide interactions [34,35]. Furthermore many FreSCOs match residues that are in close spatial proximity but at a great distance on the protein sequence (i.e. long-range interactions [32]), even though the pattern miner does not impose such a constraint. The average distance between residues matching FreSCOs is around 20 amino acids along the protein sequence chain for each rule. The distribution of these distances is distinctly bimodal [see Additional file 7], with a separation between short-range (less than 6 residues) and long-range (more than 10 residues) interactions. Most FreSCOs have an equal amount of short-range and long-range matches, which is to be expected, as the mined patterns do not consider the distance along the protein chain when evaluating best matches.

### Specific patterns correlate with optimal growth temperature

Many of the FreSCOs can be linked to interactions that contribute to the stability of the protein structure. This corresponds to previous studies on smaller data sets where similar observations have been made [21]. As mentioned before, thermophilic organisms are known to contain specific adaptations in their protein structures to stabilize them at higher temperatures. Common adaptations for thermophilic proteins include more compact proteins with a larger number of stabilizing contacts [37-39] and increased preferences for specific amino acids. If the FreSCOs are indeed critical building blocks to the overall stability of the protein structure, some should play an important role at higher temperature. To this end, the relation of the average cohesion radius of the FreSCOs to the OGT of the organism of origin was studied.

**Figure 4:**
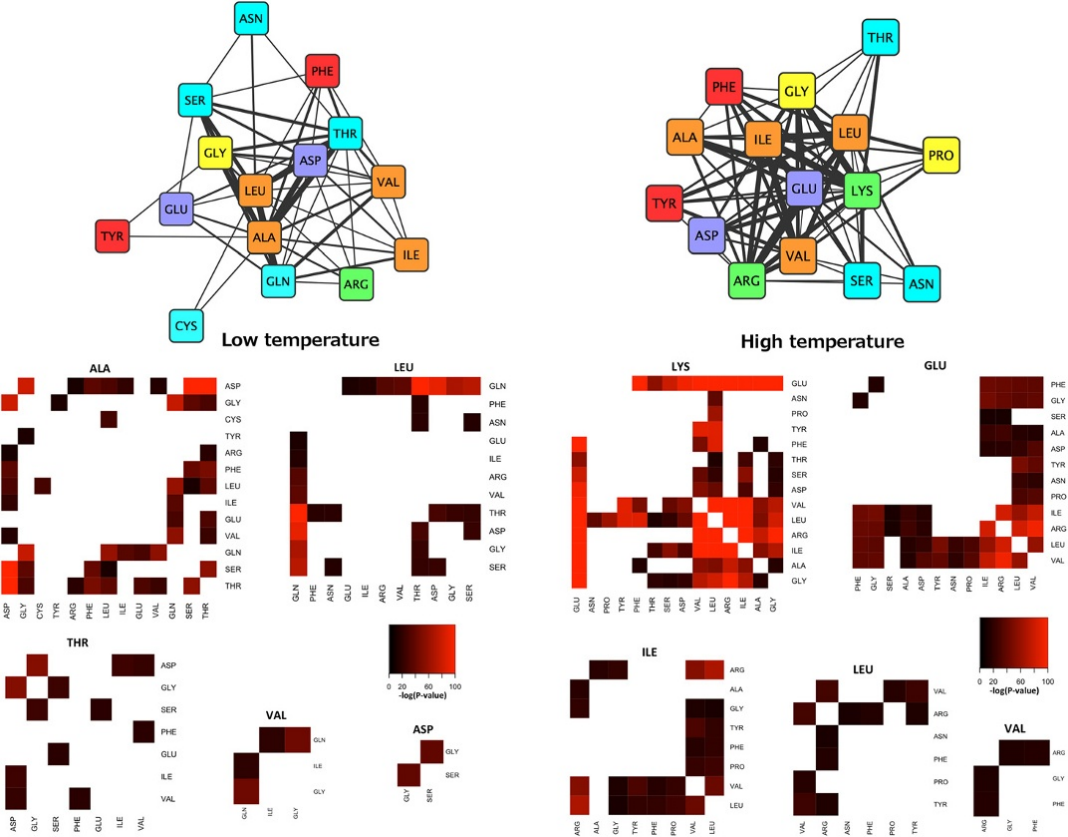
**Overview of patterns found to be associated with higher or lower OGT.** Triplet FreSCOs found to be related to proteins present in organisms with a lower (left) or higher (right) OGT. At the top of the figure is a graph where each node represents an amino acid and each edge represents patterns that include the two connecting amino acids where the edge width is scaled to the number of patterns. The nodes are colored based on the properties of the amino acid: aliphatic (orange), aromatic (red), polar (blue), acidic (purple), basic (green) and glycine/proline (yellow). At the bottom of the figure are heatmaps featuring the amino acid combinations that are either enriched in lower (left) or higher (right) OGT. The color of the heatmaps corresponds to the logarithm of the correlation p-value. The heatmaps are plotted so that each pattern occurs exactly once.

Starting from the 260 triplet FreSCOs described previously, we find that 49 were significantly correlated with lower temperatures and 95 with higher temperatures [see Additional file 8]. The most striking observation about these patterns, as can be seen in Figure 4, is that those associated with low or high OGT have similar amino acid content. For example, in both cases there are several FreSCOs that include glycine, leucine and alanine. It is already well known that IVYWREL amino acid content of the proteome increases with the OGT [28,29,40,41] and this remains true for the protein structures studied here [see Additional file 9]. It is therefore interesting that we still find many FreSCOs featuring these amino acids correlated with lower temperature. This indicates that the FreSCOs correlated with high OGT are not only determined by an increase in the frequency of these amino acids, but also due to a deliberate grouping of these residues. There are however still some differences in FreSCO residue content. For example, there are no patterns with proline or lysine correlated with low OGT and no patterns with cysteine or glutamine with high OGT. Further there are more patterns with polar residues for low OGT, and more with hydrophobic, acidic and basic residues for high OGT. This matches previous observations that as the OGT increases, the number of polar residues decreases and the number of charged and hydrophobic residues increases [42-45]. These charged residues can then form ionic bonds that stabilize the protein at higher temperatures, typically on the protein surface rather than the protein core [46-48]. The FreSCOs support this locational preference as can be seen in the strong correlation of several patterns featuring glutamate, lysine and a hydrophilic residue with high OGT. Glutamate and lysine are known to frequently occur on the protein surface as their long side chains reduce the chance of charge burial [42,43,46]. A hydrophilic amino acid (e.g. SER, ASN or THR) in the patterns thus provides the necessary context for these interactions as occurring on the protein surface.

### Mined patterns can represent preferential DNA-protein contacts

**Figure 5:**
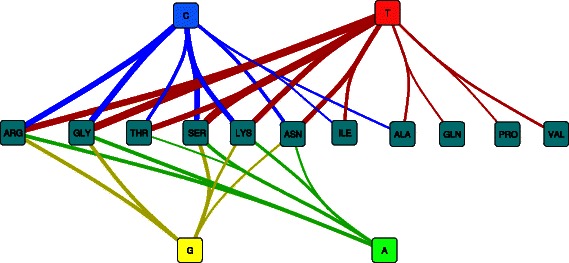
**Significant FreSCOs found in the protein-DNA complex molecular structures.** Only the associations between the bases and amino acids are shown, edges between bases or between amino acids were removed. Nodes and edges are colored by base (A: green, C: blue, G: yellow and T: red). Edge width corresponds to the number of patterns shared between the connected nodes.

**Figure 6:**
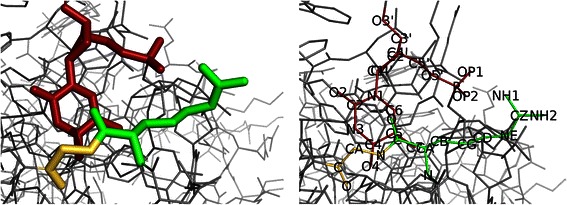
**Example of a T-GLY-ARG pattern match.** Residues matching the FreSCO T-GLY-ARG in the molecular structure of the DNA-binding cold shock protein from *Bacillus caldolyticus* [PDB:2HAX] [54]. Different subunits of the pattern have been coloured: thymine nucleotide (red), glycine (yellow) and arginine (green). Remainder of the protein and DNA-molecule have been coloured grey. The left part of the figure features the pattern in stick form, right features the labels of the non-hydrogen atoms in the pattern.

Many of the described FreSCOs were significantly enriched in nucleotide-binding proteins [see Additional file 1]. In previous analyses, the pattern miner ignored any DNA molecules that were present in the PDB molecular structures. However, patterns that combine amino acids and DNA bases can be uncovered using the spatial miner by inclusion of these molecules. FreSCOs found through such an analysis thus detail the significantly frequent and cohesive interactions between DNA-binding proteins and their ligands. These interactions are typically mediated by contacts made between amino acids and the DNA bases or backbone. It is well established that there is no clear deterministic recognition code between amino acids and bases that exists over all DNA-binding proteins [49]. However it is known that certain amino acid – base patterns exist that, while they have no predictive power, are enriched in DNA-protein complexes [50,51]. These preferential contacts between amino acids and DNA bases can be complex, featuring several bases or amino acids [52]. Therefore we will search for FreSCOs in a set of protein-DNA complexes from the PDB database in a similar manner to the patterns with only amino acids described previously. The maximum cohesive radius is increased to 7 Å, as the FreSCOs now need to bridge two macromolecules, and the support is set at 0.70. As we are interested in the contact between the protein and the DNA molecule, the FreSCOs are filtered so that only those that feature at least one base and at least one amino acid are retained. This results in 221 FreSCOs, which are combinations of one to three bases and one to two amino acids. Based on a background distribution, 94 of these patterns were found to be significant [see Additional file 10]. As can be seen in Figure 5, thymine is the base that is featured in the most FreSCOs (62 out of the 94). One possible explanation for this fact is that thymine has been found to have the largest contribution to the area surface in DNA-protein interfaces [53]. The most common amino acid in the FreSCOs is arginine (23 out of the 94). This matches previous findings where arginine is one of the primary amino acids to support interactions with DNA bases (ACGT) and the DNA backbone [34,35]. This is due to the positive charge that the arginine can carry, which allows favorable interactions with the typically negatively charged DNA molecule. Indeed, it has been observed that the DNA-protein interface is strongly enriched for the acidic amino acids arginine and lysine [53]. Other amino acids that are known to form a significant amount of the hydrogen bonds between the protein and the DNA molecule are serine, threonine, and glycine [34]. These amino acids (SER, THR, GLY, ARG, LYS) are the most represented residues among the mined FreSCOs. Amino acids that have been previously described as uncommon in protein-DNA interactions [52], such as cysteine, methionine and tryptophan, are not featured in any of the FreSCOs. The most significant FreSCO, i.e. the pattern that is most independent from the frequency of its residues or bases, was T-GLY-ARG with a p-value of 2.97 · 10^−283^. The interesting aspect of this FreSCO is the presence of glycine, which only has a small uncharged side chain, yet has been previously described as forming a significant number of hydrogen bridges in protein-DNA complexes [34]. One can easily envision an interaction where both amino acids cooperate by either forming hydrogen bridges with a thymine base, or where one amino acid stabilizes the interaction by e.g. associating with the DNA backbone. An example can be found in Figure 6 for the DNA-protein complex of the *Bacillus caldolyticus* cold shock protein (PDB: 2HAX). Here the glycine residue is in a position to directly interact with the thymine base, while the typically positively charged guanidine group of the arginine residue can interact with the negatively charged phosphate DNA backbone of the nucleotide.

## Conclusions

In this paper, we describe the mining of an interesting and relevant new pattern class, which we term ‘FreSCOs’, in the largest data set of protein molecular structures that is currently available. The found FreSCOs consist of combinations of two or three amino acids and seem to represent common building blocks aiding in the stability of the protein structure. These patterns are not directly related to any specific type of protein function as they occurred in over 90% of the analyzed structures, nor are they limited to the conserved domains of the proteins. Many of the discovered FreSCOs feature combinations of glutamate and lysine. These FreSCOS are only significant in the triplet combinations and not in the doublet patterns, suggesting an essential synergetic relationship with a third amino acid, which was often an aliphatic residue. FreSCOs featuring glutamate and lysine are found to occur mostly outside of known conserved protein domains yet are a frequent feature in protein structures. Lastly they are highly enriched in organisms that live at higher temperatures, indicating an important role in the thermostability of proteins. In general, comparison to growth temperatures reveal that hydrophilic residues were mostly found to be related with low temperatures, and hydrophobic, acidic and basic residues to high temperatures. Studying these temperature relationships with FreSCOs allows description of their synergistic tendencies among different amino acids and provides some indication of positional context, as was seen for FreSCOs containing glutamate and lysine in combination with hydrophobic residues. Further the enrichment of specific FreSCOs at higher temperature supports earlier conclusions that the mined patterns play a critical role in protein structure stability. Inclusions of the DNA molecular structures into the data set allowed description of patterns related to the contact preferences of protein-DNA complexes. Here the majority of the found patterns involve a thymine base or an arginine residue, which matches known preferences. Many of the FreSCOs described in this case indicate complex interactions involving several bases or amino acids and thus go beyond a simple one amino acid to one base contact preference.

The next step will be to extend the framework to account for the similarity between amino acids when mining these proximal patterns. The current implementation considers each amino acid type as a discrete entity, however it is well established that some amino acids are more similar than others. This can be directly integrated into the mining framework to improve the detection of important amino acid patterns. In addition, currently around half of all matches for a given FreSCO describe short-range interactions. The proposed cohesive miner can theoretically be constrained to exclude residue matches that exist in close proximity on the protein chain, which may result in more interesting and less evident patterns. Further the function analysis of the found FreSCOs supports their use in studies targeting a specific subset of proteins to discover common patterns that may exist beyond the amino acid singleton or duplet.

## Additional files

Additional file 1:
**Patterns mined from protein structure sets with lower redundancy.**
Additional file 2:
**Example of the cohesion radius distribution for permutated protein structures.**
Additional file 3:
**Frequency and cohesion radius of the significant doublet and triplet FreSCOs in the non-redundant protein data set.**
Additional file 4:
**Domain enrichment and depletion P-values for each mined FreSCO.**
Additional file 5:
**Domain enrichment and depletion P-values of individual amino acids.**
Additional file 6:
**Gene ontology enrichment for the 104 significant FreSCOs.**
Additional file 7:
**Distribution of the distance along the protein sequence between residues captured for each FreSCO match.**
Additional file 8:
**Mined FreSCOs significantly correlated with optimal growth temperature.**
Additional file 9:
**Correlation of individual amino acids in a protein and the associated OGT.**
Additional file 10:
**Overview of the significance of the found amino acid–base FreSCOs in the protein-DNA complex structure data set.**

## Abbreviations

FreSCO: Frequent spatially cohesive component set
OGT: Optimal growth temperature
PCM: Protein contact map
PDB: RCSB Protein DataBank
