# Supplementary material for: Mining the entire Protein DataBank for frequent spatially cohesive amino acid patterns
Source: BioData Min. 2015 Jan 31;8:4. doi: 10.1186/s13040-015-0038-4 (PMC4318390; doi:10.1186/s13040-015-0038-4)

Additional file 10: Patterns mined from protein structure sets with lower redundancy.

Three sets of protein molecular structures were obtained from PDB, each with a different degree of redundancy. The non-redundant set of protein chains was obtained from the nr-PDB collection on the VAST website and is based on a maximal BLAST similarity p-value of either $1 \cdot 10^{-80}$ (32 142 proteins), $1 \cdot 10^{-40}$ (22 694 proteins), or $1 \cdot 10^{-7}$ (13 176 proteins). Higher p-value cutoffs signify lower redundancy, as more protein structures will be excluded. Each set of structures was mined for FreSCOs with a maximum cohesive radius of 4.5 Å and a minimal support of 0.60. As can be seen in table 10.1, the results for the cohesion and frequency of a given FreSCO are highly similar between the data sets and are therefore largely independent of the redundancy in the data set. For example, the Pearson correlation between the cohesion radius of a FreSCO in the 1E-80 set and the 1E-7 set is 0.9990. The significance of each found pattern was separately then independently estimated as described in the materials and methods against the data set of origin. So the cohesion radius of a FreSCO from the 1E-7 data set is compared against the cohesion radiuses from several permutated versions of the same data set. This results in three lists of FreSCOs, one for each data set, which have cohesion radiuses that are significantly smaller than those from the permutated sets. As can be seen in figure 10.1, almost all of these significant FreSCOs are shared in the result from all three data sets. The assigned P-values were also highly correlated among the shared significant FreSCOs. For example, the Spearman rank correlation between the P-values from the 1E-80 and 1E-7 FreSCOs was 0.7126.

Table 10.1: Cohesive radius and frequency for FreSCOs mined with a cohesion radius < 4.5 Å and a support > 0.60 in all three data sets. The single amino acid FreSCOs have also been included here to illustrate the difference in amino acid frequency between the data sets.

FreSCO	1E-80 redundancy		1E-40 redundancy		1E-7 redundancy	
	Cohesion	Frequency	Cohesion	Frequency	Cohesion	Frequency
ILE	0.000	0.964	0.000	0.954	0.000	0.948
VAL	0.000	0.970	0.000	0.958	0.000	0.954
TYR	0.000	0.933	0.000	0.915	0.000	0.904
MET	0.000	0.721	0.000	0.676	0.000	0.650
ALA	0.000	0.981	0.000	0.974	0.000	0.969
HIS	0.000	0.871	0.000	0.851	0.000	0.852
LYS	0.000	0.974	0.000	0.967	0.000	0.960
LEU	0.000	0.987	0.000	0.982	0.000	0.976
ARG	0.000	0.969	0.000	0.962	0.000	0.959
CYS	0.000	0.715	0.000	0.695	0.000	0.690
ASP	0.000	0.968	0.000	0.955	0.000	0.948
ASN	0.000	0.956	0.000	0.942	0.000	0.932
GLY	0.000	0.979	0.000	0.972	0.000	0.966
SER	0.000	0.977	0.000	0.972	0.000	0.970

GLU	0.000	0.978	0.000	0.970	0.000	0.962
PRO	0.000	0.946	0.000	0.930	0.000	0.928
GLN	0.000	0.954	0.000	0.941	0.000	0.933
PHE	0.000	0.943	0.000	0.923	0.000	0.920
THR	0.000	0.970	0.000	0.958	0.000	0.951
TRP	0.000	0.739	0.000	0.719	0.000	0.728
ALA-LEU	2.206	0.972	2.246	0.961	2.225	0.952
LEU-VAL	2.214	0.962	2.253	0.947	2.239	0.941
GLU-LEU	2.223	0.970	2.248	0.959	2.226	0.949
LEU-LYS	2.236	0.964	2.275	0.953	2.258	0.943
LEU-SER	2.288	0.967	2.332	0.958	2.298	0.953
GLY-LEU	2.295	0.968	2.339	0.956	2.321	0.947
ILE-LEU	2.309	0.956	2.335	0.944	2.292	0.936
LEU-THR	2.310	0.961	2.368	0.946	2.344	0.936
ALA-VAL	2.325	0.957	2.374	0.940	2.374	0.934
ALA-GLU	2.326	0.964	2.354	0.950	2.349	0.940
ASP-LEU	2.337	0.960	2.379	0.944	2.322	0.934
ARG-LEU	2.337	0.960	2.355	0.949	2.318	0.943
GLU-VAL	2.339	0.954	2.365	0.937	2.355	0.930
LYS-VAL	2.372	0.949	2.437	0.934	2.439	0.925
ALA-LYS	2.375	0.959	2.432	0.947	2.422	0.936
GLU-LYS	2.376	0.957	2.409	0.944	2.407	0.932
GLU-GLY	2.383	0.961	2.423	0.946	2.427	0.935
ILE-VAL	2.404	0.943	2.461	0.925	2.454	0.920
GLY-LYS	2.420	0.955	2.483	0.942	2.514	0.932
ALA-ILE	2.422	0.951	2.469	0.937	2.429	0.928
ALA-GLY	2.424	0.964	2.486	0.951	2.492	0.942
GLY-VAL	2.434	0.956	2.510	0.940	2.542	0.933
GLU-SER	2.455	0.959	2.479	0.947	2.466	0.941
ALA-SER	2.457	0.962	2.497	0.951	2.469	0.947
ASP-VAL	2.459	0.948	2.514	0.927	2.494	0.920
GLY-SER	2.460	0.960	2.504	0.949	2.477	0.944
THR-VAL	2.462	0.949	2.534	0.930	2.525	0.923
GLN-LEU	2.466	0.946	2.498	0.930	2.449	0.920
ARG-VAL	2.471	0.945	2.516	0.928	2.502	0.925
LEU-PHE	2.471	0.936	2.482	0.915	2.451	0.910
GLU-ILE	2.473	0.949	2.479	0.934	2.450	0.924
SER-VAL	2.473	0.953	2.512	0.939	2.499	0.936
ASN-LEU	2.475	0.947	2.514	0.931	2.479	0.918
ALA-ASP	2.478	0.956	2.544	0.938	2.511	0.928
ARG-GLU	2.482	0.953	2.475	0.939	2.447	0.931
GLU-THR	2.491	0.954	2.526	0.937	2.498	0.926
ALA-THR	2.511	0.956	2.583	0.940	2.550	0.931
ALA-ARG	2.528	0.955	2.555	0.943	2.541	0.937
LEU-TYR	2.531	0.926	2.553	0.905	2.500	0.892

ASP-GLU	2.534	0.955	2.526	0.938	2.510	0.929
LEU-PRO	2.544	0.935	2.547	0.916	2.501	0.909
ASP-LYS	2.545	0.949	2.580	0.932	2.589	0.921
GLY-ILE	2.547	0.948	2.592	0.933	2.593	0.925
ASP-GLY	2.548	0.953	2.638	0.935	2.608	0.926
GLY-THR	2.556	0.955	2.630	0.939	2.661	0.929
ILE-LYS	2.565	0.944	2.607	0.930	2.596	0.920
ARG-GLY	2.572	0.952	2.615	0.939	2.626	0.933
ARG-ILE	2.573	0.940	2.592	0.924	2.573	0.919
ILE-THR	2.574	0.943	2.647	0.926	2.639	0.917
PHE-VAL	2.583	0.925	2.627	0.899	2.638	0.896
ILE-SER	2.587	0.946	2.626	0.933	2.597	0.929
LYS-SER	2.597	0.956	2.627	0.946	2.626	0.939
LYS-THR	2.600	0.949	2.679	0.934	2.683	0.923
ASP-SER	2.603	0.951	2.643	0.936	2.611	0.930
ASP-ILE	2.605	0.942	2.646	0.923	2.602	0.914
SER-THR	2.645	0.953	2.700	0.939	2.671	0.933
ARG-LYS	2.646	0.948	2.671	0.936	2.630	0.928
ALA-PHE	2.647	0.931	2.655	0.908	2.639	0.902
ASP-THR	2.655	0.946	2.734	0.926	2.700	0.917
ARG-ASP	2.661	0.945	2.712	0.927	2.675	0.921
GLU-PHE	2.666	0.930	2.677	0.906	2.662	0.900
ALA-ASN	2.678	0.943	2.732	0.926	2.717	0.913
ALA-GLN	2.682	0.942	2.732	0.924	2.673	0.915
GLN-VAL	2.682	0.934	2.729	0.913	2.709	0.906
TYR-VAL	2.689	0.914	2.740	0.889	2.732	0.880
ASN-VAL	2.693	0.936	2.762	0.916	2.765	0.905
ARG-SER	2.697	0.952	2.715	0.941	2.658	0.937
PRO-VAL	2.701	0.927	2.741	0.905	2.733	0.901
GLY-PRO	2.714	0.935	2.778	0.916	2.797	0.911
ALA-PRO	2.724	0.932	2.772	0.912	2.761	0.906
GLY-PHE	2.729	0.931	2.791	0.908	2.805	0.902
ARG-THR	2.735	0.946	2.795	0.930	2.756	0.923
ASN-GLU	2.740	0.943	2.752	0.925	2.721	0.913
HIS-LEU	2.742	0.867	2.737	0.844	2.697	0.844
GLN-GLU	2.742	0.941	2.736	0.923	2.677	0.913
ALA-TYR	2.757	0.922	2.783	0.899	2.752	0.887
CYS-LEU	2.774	0.708	2.784	0.685	2.771	0.678
PRO-SER	2.774	0.930	2.778	0.912	2.752	0.909
GLU-PRO	2.778	0.931	2.735	0.909	2.717	0.903
PHE-SER	2.780	0.928	2.797	0.906	2.787	0.905
ASN-GLY	2.784	0.940	2.869	0.922	2.869	0.910
ILE-PHE	2.791	0.919	2.849	0.896	2.812	0.891
LYS-PHE	2.804	0.924	2.845	0.901	2.865	0.894
GLU-TYR	2.816	0.921	2.814	0.898	2.782	0.886

LYS-PRO	2.819	0.926	2.864	0.906	2.891	0.899
GLN-GLY	2.832	0.939	2.935	0.920	2.887	0.910
GLN-ILE	2.833	0.928	2.881	0.909	2.840	0.899
GLN-LYS	2.849	0.935	2.890	0.918	2.857	0.906
ASN-ILE	2.852	0.929	2.918	0.910	2.902	0.899
GLY-TYR	2.857	0.922	2.924	0.899	2.919	0.887
PHE-THR	2.862	0.926	2.912	0.901	2.914	0.896
ILE-TYR	2.863	0.910	2.905	0.887	2.870	0.877
ASP-PHE	2.864	0.924	2.880	0.897	2.844	0.893
ASP-PRO	2.868	0.924	2.919	0.900	2.895	0.895
ASN-LYS	2.869	0.938	2.941	0.921	2.937	0.906
PRO-THR	2.873	0.928	2.956	0.907	2.955	0.900
ARG-PHE	2.883	0.922	2.885	0.899	2.870	0.897
LEU-MET	2.890	0.716	2.944	0.669	2.919	0.642
LYS-TYR	2.913	0.915	2.979	0.894	2.970	0.881
ASN-ASP	2.925	0.936	3.016	0.915	2.976	0.904
ARG-ASN	2.930	0.933	2.964	0.915	2.924	0.905
GLN-SER	2.936	0.937	2.961	0.921	2.899	0.914
ASN-SER	2.939	0.940	2.993	0.924	2.958	0.915
ILE-PRO	2.944	0.918	2.958	0.896	2.925	0.891
SER-TYR	2.948	0.918	2.983	0.897	2.950	0.888
ARG-GLN	2.968	0.931	2.994	0.914	2.920	0.906
ASP-TYR	2.970	0.915	3.019	0.890	2.975	0.879
LEU-TRP	2.980	0.734	2.976	0.713	2.931	0.722
CYS-VAL	2.980	0.702	3.007	0.676	3.053	0.672
ASN-THR	2.981	0.935	3.096	0.914	3.056	0.902
GLN-THR	2.983	0.932	3.048	0.911	3.000	0.901
ARG-TYR	2.987	0.913	2.993	0.891	2.938	0.882
ASP-GLN	2.987	0.932	3.036	0.910	2.961	0.901
THR-TYR	3.004	0.916	3.080	0.891	3.052	0.880
ALA-CYS	3.005	0.706	3.032	0.682	3.087	0.675
ARG-PRO	3.023	0.925	3.060	0.905	3.053	0.902
ALA-HIS	3.050	0.862	3.068	0.838	3.056	0.838
HIS-VAL	3.069	0.857	3.099	0.832	3.099	0.833
CYS-GLY	3.133	0.710	3.160	0.688	3.260	0.682
ALA-MET	3.139	0.713	3.211	0.665	3.169	0.639
MET-VAL	3.154	0.709	3.211	0.660	3.233	0.634
GLN-PHE	3.164	0.908	3.199	0.881	3.175	0.874
ASN-PHE	3.170	0.912	3.227	0.886	3.209	0.879
GLU-HIS	3.173	0.863	3.108	0.839	3.058	0.837
PHE-TYR	3.184	0.895	3.179	0.865	3.147	0.857
ASN-PRO	3.204	0.912	3.243	0.887	3.207	0.878
GLY-HIS	3.212	0.861	3.232	0.837	3.240	0.837
HIS-ILE	3.220	0.850	3.211	0.825	3.196	0.828
PHE-PRO	3.224	0.907	3.194	0.880	3.195	0.875

GLU-MET	3.233	0.712	3.218	0.664	3.226	0.637
TRP-VAL	3.234	0.729	3.254	0.706	3.231	0.715
CYS-ILE	3.276	0.695	3.275	0.670	3.279	0.664
CYS-GLU	3.279	0.705	3.267	0.681	3.270	0.674
LYS-MET	3.294	0.709	3.373	0.662	3.382	0.634
HIS-SER	3.299	0.861	3.259	0.840	3.235	0.841
ALA-TRP	3.302	0.732	3.319	0.710	3.290	0.718
PRO-TYR	3.305	0.896	3.328	0.869	3.307	0.860
GLN-PRO	3.306	0.910	3.359	0.885	3.295	0.879
ASN-GLN	3.316	0.922	3.353	0.901	3.305	0.887
CYS-SER	3.328	0.707	3.344	0.685	3.345	0.681
ILE-MET	3.333	0.704	3.434	0.656	3.404	0.630
HIS-LYS	3.339	0.857	3.386	0.834	3.366	0.832
GLY-MET	3.340	0.711	3.416	0.663	3.382	0.636
GLN-TYR	3.348	0.901	3.401	0.874	3.312	0.863
ASN-TYR	3.374	0.905	3.420	0.879	3.380	0.866
GLU-TRP	3.380	0.732	3.334	0.711	3.290	0.718
CYS-THR	3.388	0.705	3.440	0.680	3.469	0.673
MET-SER	3.390	0.710	3.436	0.664	3.369	0.640
GLY-TRP	3.396	0.732	3.443	0.711	3.462	0.719
HIS-THR	3.401	0.857	3.431	0.830	3.421	0.831
ASP-HIS	3.448	0.856	3.443	0.829	3.357	0.830
ARG-HIS	3.471	0.857	3.446	0.835	3.384	0.835
ASP-CYS	3.472	0.701	3.465	0.675	3.475	0.670
ASP-MET	3.480	0.709	3.533	0.659	3.490	0.630
MET-THR	3.519	0.707	3.589	0.657	3.577	0.631
ALA-LEU-VAL	3.520	0.950	3.586	0.932	3.562	0.924
ILE-TRP	3.527	0.724	3.544	0.703	3.513	0.709
LYS-TRP	3.531	0.726	3.552	0.704	3.528	0.710
CYS-LYS	3.533	0.702	3.571	0.679	3.604	0.671
ARG-CYS	3.534	0.701	3.526	0.679	3.530	0.672
ARG-MET	3.558	0.705	3.570	0.658	3.549	0.633
SER-TRP	3.563	0.728	3.580	0.707	3.514	0.719
ALA-GLU-LEU	3.570	0.958	3.598	0.942	3.560	0.930
CYS-PHE	3.593	0.691	3.586	0.663	3.637	0.657
ASP-TRP	3.604	0.730	3.605	0.706	3.571	0.715
GLU-LEU-VAL	3.613	0.949	3.646	0.930	3.619	0.922
ARG-TRP	3.634	0.726	3.614	0.704	3.550	0.714
ALA-LEU-LYS	3.636	0.951	3.687	0.936	3.663	0.924
GLU-LEU-LYS	3.650	0.950	3.679	0.935	3.651	0.922
ALA-ILE-LEU	3.652	0.945	3.692	0.928	3.631	0.919
ILE-LEU-VAL	3.669	0.938	3.722	0.918	3.686	0.912
HIS-PHE	3.675	0.840	3.628	0.810	3.615	0.814
LEU-LYS-VAL	3.700	0.943	3.738	0.925	3.712	0.914
THR-TRP	3.700	0.728	3.713	0.706	3.685	0.713

ALA-GLY-LEU	3.728	0.956	3.796	0.940	3.780	0.928
ALA-LEU-SER	3.757	0.955	3.808	0.941	3.752	0.934
GLU-ILE-LEU	3.758	0.944	3.755	0.927	3.706	0.917
GLY-LEU-VAL	3.765	0.949	3.840	0.930	3.835	0.922
ARG-GLU-LEU	3.776	0.947	3.776	0.931	3.710	0.922
LEU-THR-VAL	3.785	0.943	3.853	0.922	3.812	0.913
ALA-ARG-LEU	3.785	0.948	3.813	0.933	3.765	0.925
ALA-LEU-THR	3.786	0.949	3.856	0.930	3.800	0.919
ALA-GLU-VAL	3.789	0.944	3.825	0.923	3.826	0.915
LEU-SER-VAL	3.790	0.947	3.831	0.930	3.795	0.926
ARG-LEU-VAL	3.794	0.939	3.821	0.921	3.777	0.915
ALA-ASP-LEU	3.803	0.949	3.860	0.929	3.786	0.918
HIS-PRO	3.805	0.841	3.763	0.813	3.747	0.816
ALA-ILE-VAL	3.811	0.933	3.874	0.911	3.865	0.906
ASP-LEU-VAL	3.812	0.942	3.857	0.920	3.793	0.911
MET-PHE	3.813	0.692	3.885	0.639	3.906	0.617
GLU-LEU-SER	3.818	0.953	3.847	0.939	3.797	0.931
GLU-GLY-LEU	3.818	0.954	3.863	0.937	3.837	0.925
ASN-HIS	3.822	0.847	3.855	0.820	3.843	0.818
HIS-TYR	3.845	0.832	3.829	0.803	3.807	0.802
ILE-LEU-LYS	3.845	0.937	3.884	0.921	3.843	0.909
CYS-PRO	3.846	0.698	3.829	0.674	3.887	0.667
GLU-LEU-THR	3.847	0.948	3.898	0.929	3.846	0.917
GLY-LEU-LYS	3.853	0.947	3.924	0.931	3.934	0.917
ALA-GLU-LYS	3.868	0.945	3.933	0.928	3.920	0.914
ALA-LYS-VAL	3.888	0.938	3.967	0.919	3.971	0.909
CYS-TYR	3.893	0.687	3.885	0.660	3.894	0.650
GLU-LYS-VAL	3.894	0.937	3.940	0.917	3.928	0.907
ARG-ILE-LEU	3.894	0.934	3.914	0.917	3.851	0.910
GLY-ILE-LEU	3.895	0.942	3.945	0.924	3.911	0.914
ILE-LEU-THR	3.899	0.937	3.966	0.918	3.929	0.908
ASP-LEU-LYS	3.899	0.942	3.939	0.923	3.899	0.910
ILE-LEU-SER	3.909	0.940	3.942	0.925	3.889	0.919
ALA-LEU-PHE	3.918	0.926	3.935	0.902	3.890	0.895
PHE-TRP	3.918	0.719	3.922	0.693	3.902	0.701
ASN-CYS	3.919	0.695	3.974	0.670	4.004	0.663
GLY-LEU-SER	3.921	0.951	3.988	0.937	3.943	0.930
ASP-GLU-LEU	3.922	0.949	3.914	0.929	3.867	0.920
GLN-HIS	3.923	0.845	3.910	0.819	3.844	0.818
LEU-PHE-VAL	3.926	0.921	3.952	0.893	3.932	0.889
ALA-GLY-VAL	3.939	0.944	4.016	0.924	4.039	0.916
ALA-GLU-ILE	3.941	0.938	3.953	0.919	3.915	0.909
ASN-MET	3.945	0.699	4.065	0.649	3.983	0.620
LEU-LYS-SER	3.952	0.947	3.990	0.934	3.954	0.925
ASP-ILE-LEU	3.953	0.937	3.992	0.916	3.917	0.906

GLN-MET	3.955	0.698	3.975	0.648	3.902	0.620
LEU-LYS-THR	3.955	0.941	4.033	0.923	4.007	0.910
ALA-GLN-LEU	3.965	0.936	4.006	0.916	3.927	0.905
GLU-ILE-VAL	3.971	0.931	3.951	0.909	3.931	0.903
ARG-GLY-LEU	3.972	0.945	4.027	0.928	3.992	0.920
GLY-LEU-THR	3.975	0.947	4.056	0.928	4.040	0.917
ARG-LEU-LYS	3.981	0.940	4.008	0.925	3.954	0.916
ALA-SER-VAL	3.989	0.942	4.044	0.924	4.030	0.920
ALA-THR-VAL	3.992	0.939	4.075	0.916	4.052	0.909
MET-PRO	3.994	0.690	3.986	0.638	3.989	0.614
ALA-ARG-VAL	4.001	0.935	4.049	0.914	4.036	0.909
ASP-LEU-SER	4.002	0.945	4.045	0.926	3.965	0.920
ALA-ASP-VAL	4.002	0.938	4.078	0.915	4.053	0.906
LEU-TYR-VAL	4.005	0.910	4.055	0.882	3.996	0.873
CYS-GLN	4.012	0.691	4.052	0.664	4.032	0.659
ALA-LEU-TYR	4.012	0.916	4.043	0.891	3.979	0.878
ASP-GLY-LEU	4.018	0.946	4.108	0.925	4.039	0.914
ALA-ASN-LEU	4.018	0.936	4.067	0.916	4.024	0.903
ARG-ASP-LEU	4.019	0.939	4.046	0.919	3.971	0.912
GLU-LEU-PHE	4.021	0.926	4.021	0.900	3.989	0.894
GLN-LEU-VAL	4.023	0.928	4.062	0.905	4.008	0.897
ALA-ARG-GLU	4.023	0.941	4.037	0.924	4.009	0.915
GLU-GLY-VAL	4.024	0.943	4.092	0.922	4.108	0.913
ALA-ILE-LYS	4.024	0.933	4.090	0.915	4.073	0.903
LEU-SER-THR	4.030	0.946	4.079	0.929	4.024	0.922
ARG-GLU-VAL	4.035	0.933	4.017	0.912	3.980	0.907
GLY-ILE-VAL	4.039	0.932	4.103	0.911	4.132	0.904
ILE-LYS-VAL	4.046	0.926	4.125	0.905	4.134	0.898
ALA-GLU-GLY	4.052	0.949	4.107	0.930	4.106	0.918
PRO-TRP	4.053	0.721	3.998	0.696	4.018	0.706
MET-TYR	4.056	0.681	4.145	0.627	4.099	0.600
GLU-ILE-LYS	4.057	0.931	4.091	0.913	4.072	0.901
ASP-LEU-THR	4.060	0.940	4.126	0.917	4.053	0.907
ILE-THR-VAL	4.061	0.927	4.150	0.904	4.159	0.899
ALA-GLY-ILE	4.062	0.937	4.133	0.918	4.131	0.908
GLU-GLY-LYS	4.070	0.941	4.152	0.923	4.169	0.909
ARG-LEU-SER	4.074	0.945	4.085	0.931	4.010	0.925
GLN-GLU-LEU	4.075	0.936	4.066	0.916	3.988	0.905
ARG-LEU-THR	4.075	0.939	4.137	0.920	4.080	0.912
ALA-GLU-SER	4.081	0.947	4.103	0.930	4.074	0.923
ASN-LEU-VAL	4.084	0.930	4.140	0.908	4.091	0.896
ALA-PHE-VAL	4.085	0.916	4.114	0.887	4.118	0.883
ASN-TRP	4.086	0.719	4.130	0.695	4.086	0.701
ALA-GLY-LYS	4.088	0.943	4.182	0.926	4.212	0.913
ALA-GLU-THR	4.093	0.943	4.153	0.922	4.110	0.911

GLY-LYS-VAL	4.098	0.937	4.186	0.917	4.239	0.907
ALA-ARG-ILE	4.099	0.929	4.140	0.911	4.123	0.904
ILE-LEU-PHE	4.099	0.915	4.126	0.890	4.070	0.885
ARG-ILE-VAL	4.100	0.922	4.129	0.900	4.128	0.897
GLN-TRP	4.104	0.719	4.116	0.695	4.011	0.702
GLU-THR-VAL	4.104	0.937	4.129	0.915	4.098	0.906
GLU-SER-VAL	4.106	0.941	4.107	0.922	4.080	0.917
ASP-ILE-VAL	4.122	0.927	4.188	0.903	4.164	0.896
TRP-TYR	4.122	0.713	4.167	0.687	4.095	0.692
ILE-SER-VAL	4.127	0.929	4.179	0.910	4.146	0.908
GLY-THR-VAL	4.128	0.938	4.223	0.916	4.256	0.907
ALA-ILE-THR	4.130	0.932	4.204	0.911	4.183	0.902
ALA-ASP-ILE	4.131	0.932	4.199	0.911	4.159	0.900
ALA-GLY-SER	4.132	0.947	4.200	0.932	4.176	0.925
GLY-SER-VAL	4.134	0.942	4.216	0.924	4.230	0.919
ARG-GLU-ILE	4.137	0.928	4.108	0.908	4.060	0.901
ALA-ILE-SER	4.137	0.935	4.173	0.918	4.123	0.912
LEU-LYS-PHE	4.139	0.919	4.159	0.894	4.150	0.886
ASP-LYS-VAL	4.141	0.932	4.202	0.909	4.192	0.900
GLY-LEU-PHE	4.142	0.925	4.179	0.900	4.169	0.894
ASN-GLU-LEU	4.144	0.936	4.147	0.916	4.096	0.903
GLN-ILE-LEU	4.147	0.923	4.181	0.902	4.109	0.892
LEU-PRO-VAL	4.153	0.920	4.184	0.895	4.153	0.889
ALA-ASP-GLU	4.156	0.945	4.183	0.924	4.149	0.913
LEU-PHE-SER	4.160	0.923	4.165	0.900	4.133	0.898
ILE-LEU-TYR	4.161	0.905	4.190	0.880	4.110	0.869
ALA-LEU-PRO	4.164	0.925	4.182	0.902	4.145	0.893
GLU-LEU-TYR	4.165	0.916	4.165	0.892	4.091	0.879
ALA-ASP-LYS	4.166	0.938	4.223	0.918	4.215	0.904
ALA-GLY-THR	4.174	0.943	4.285	0.923	4.291	0.912
ASP-GLY-VAL	4.175	0.937	4.280	0.914	4.287	0.905
GLN-LEU-LYS	4.176	0.928	4.222	0.908	4.155	0.896
ASP-GLU-VAL	4.178	0.938	4.176	0.914	4.147	0.907
GLU-GLY-ILE	4.189	0.935	4.231	0.916	4.211	0.905
ARG-GLY-VAL	4.189	0.933	4.255	0.913	4.280	0.907
ALA-ASP-GLY	4.201	0.943	4.308	0.922	4.283	0.910
ALA-LYS-SER	4.201	0.943	4.279	0.929	4.257	0.920
LYS-THR-VAL	4.204	0.932	4.306	0.910	4.312	0.900
ASN-ILE-LEU	4.204	0.924	4.253	0.902	4.201	0.891
ILE-PHE-VAL	4.204	0.905	4.253	0.876	4.255	0.874
ALA-TYR-VAL	4.209	0.905	4.252	0.877	4.238	0.867
ARG-LEU-PHE	4.209	0.917	4.214	0.892	4.173	0.889
GLU-ILE-THR	4.217	0.932	4.254	0.911	4.230	0.901
GLU-ILE-SER	4.218	0.934	4.228	0.916	4.195	0.910
LEU-PHE-THR	4.218	0.921	4.262	0.895	4.234	0.888

GLU-PHE-VAL	4.221	0.915	4.211	0.886	4.208	0.882
ALA-ARG-GLY	4.223	0.941	4.295	0.923	4.308	0.915
GLU-LYS-SER	4.225	0.941	4.251	0.926	4.235	0.916
ALA-GLU-PHE	4.235	0.920	4.244	0.894	4.221	0.886
GLU-LYS-THR	4.238	0.936	4.299	0.916	4.283	0.903
ARG-GLU-LYS	4.239	0.935	4.236	0.917	4.196	0.907
ALA-LYS-THR	4.239	0.938	4.341	0.918	4.329	0.906
LEU-LYS-TYR	4.240	0.909	4.289	0.885	4.231	0.871
ARG-GLU-GLY	4.241	0.938	4.263	0.920	4.261	0.910
GLU-GLY-SER	4.242	0.945	4.284	0.928	4.274	0.920
ASN-LEU-LYS	4.242	0.931	4.307	0.911	4.273	0.895
LYS-SER-VAL	4.243	0.935	4.300	0.918	4.284	0.911
ASP-SER-VAL	4.250	0.934	4.306	0.913	4.269	0.908
GLY-ILE-LYS	4.250	0.929	4.322	0.911	4.350	0.899
ARG-ASP-VAL	4.251	0.927	4.314	0.904	4.264	0.899
GLY-LEU-TYR	4.253	0.915	4.320	0.890	4.265	0.877
ALA-GLN-VAL	4.253	0.924	4.307	0.900	4.266	0.893
GLU-GLY-THR	4.261	0.942	4.329	0.921	4.338	0.909
GLN-LEU-SER	4.262	0.931	4.289	0.912	4.211	0.905
ASP-LEU-PHE	4.262	0.919	4.257	0.891	4.189	0.886
ASN-GLY-LEU	4.262	0.933	4.341	0.912	4.313	0.898
ASP-GLU-LYS	4.263	0.938	4.267	0.918	4.242	0.906
SER-THR-VAL	4.264	0.936	4.328	0.915	4.298	0.911
ALA-ILE-PHE	4.265	0.910	4.293	0.884	4.251	0.878
GLY-ILE-THR	4.267	0.931	4.359	0.910	4.376	0.901
ALA-ASP-SER	4.267	0.941	4.317	0.922	4.267	0.914
GLY-PHE-VAL	4.267	0.916	4.332	0.888	4.368	0.883
ALA-SER-THR	4.272	0.941	4.343	0.923	4.306	0.917
GLU-LEU-PRO	4.274	0.924	4.250	0.900	4.203	0.892
ARG-LYS-VAL	4.276	0.928	4.331	0.908	4.313	0.901
GLY-ILE-SER	4.277	0.933	4.335	0.916	4.332	0.910
ALA-ARG-LYS	4.279	0.936	4.315	0.920	4.295	0.910
LEU-SER-TYR	4.280	0.913	4.317	0.889	4.242	0.879
ASP-THR-VAL	4.280	0.931	4.375	0.906	4.346	0.899
GLN-GLY-LEU	4.281	0.932	4.364	0.911	4.291	0.899
ALA-ARG-ASP	4.286	0.935	4.353	0.914	4.300	0.906
ARG-GLN-LEU	4.286	0.925	4.307	0.905	4.214	0.897
ASP-GLU-ILE	4.292	0.932	4.283	0.910	4.247	0.900
ASN-LEU-SER	4.295	0.933	4.353	0.915	4.287	0.905
ALA-ASP-THR	4.296	0.937	4.394	0.913	4.350	0.903
ASP-GLY-LYS	4.298	0.935	4.398	0.915	4.399	0.902
ARG-LEU-TYR	4.299	0.907	4.310	0.883	4.227	0.872
ALA-ASN-VAL	4.299	0.926	4.360	0.902	4.359	0.890
GLN-LEU-THR	4.307	0.926	4.373	0.903	4.305	0.893
ARG-GLY-ILE	4.310	0.927	4.355	0.907	4.367	0.901

ARG-ASN-LEU	4.315	0.926	4.344	0.906	4.280	0.895
ILE-TYR-VAL	4.323	0.895	4.371	0.866	4.347	0.860
ALA-GLY-PHE	4.323	0.921	4.363	0.895	4.382	0.887
ASP-ILE-LYS	4.324	0.926	4.364	0.905	4.342	0.893
ARG-THR-VAL	4.326	0.928	4.397	0.905	4.363	0.900
ASP-GLY-ILE	4.327	0.930	4.404	0.908	4.371	0.898
GLY-LEU-PRO	4.333	0.926	4.369	0.903	4.345	0.895
LEU-PRO-SER	4.334	0.922	4.348	0.901	4.294	0.896
ARG-SER-VAL	4.335	0.932	4.354	0.914	4.320	0.910
ASN-ASP-LEU	4.337	0.929	4.406	0.906	4.324	0.894
CYS-LEU-VAL	4.338	0.696	4.368	0.669	4.360	0.663
ALA-CYS-LEU	4.338	0.700	4.373	0.674	4.392	0.666
ALA-PRO-VAL	4.338	0.917	4.388	0.892	4.390	0.886
ALA-ARG-THR	4.339	0.935	4.422	0.915	4.391	0.908
GLY-LYS-SER	4.342	0.940	4.422	0.925	4.419	0.916
ASN-LEU-THR	4.345	0.928	4.428	0.905	4.364	0.892
GLY-LYS-THR	4.348	0.936	4.452	0.916	4.489	0.904
ASP-GLN-LEU	4.349	0.927	4.383	0.903	4.278	0.893
ALA-ARG-SER	4.349	0.940	4.351	0.925	4.303	0.919
ASP-GLY-SER	4.349	0.938	4.425	0.919	4.389	0.912
LEU-THR-TYR	4.349	0.910	4.409	0.884	4.337	0.871
LYS-PHE-VAL	4.349	0.909	4.424	0.881	4.444	0.875
PHE-THR-VAL	4.351	0.912	4.418	0.882	4.430	0.878
ALA-PHE-SER	4.353	0.918	4.372	0.894	4.354	0.891
ALA-GLN-GLU	4.353	0.931	4.369	0.909	4.289	0.899
GLY-SER-THR	4.356	0.940	4.438	0.922	4.444	0.915
ILE-LYS-THR	4.357	0.926	4.442	0.905	4.438	0.895
ARG-ASP-ILE	4.358	0.922	4.391	0.900	4.350	0.894
ASP-LEU-TYR	4.362	0.910	4.397	0.883	4.297	0.871
ALA-GLN-ILE	4.362	0.919	4.409	0.897	4.361	0.886
LEU-LYS-PRO	4.366	0.917	4.375	0.894	4.362	0.885
ILE-LYS-SER	4.368	0.929	4.411	0.913	4.408	0.905
ASP-GLU-GLY	4.371	0.942	4.405	0.920	4.378	0.910
HIS-LEU-VAL	4.372	0.854	4.393	0.827	4.371	0.827
PHE-SER-VAL	4.372	0.913	4.406	0.887	4.405	0.886
ILE-SER-THR	4.375	0.929	4.438	0.910	4.418	0.905
GLU-LYS-PHE	4.377	0.914	4.383	0.887	4.391	0.879
ALA-ILE-TYR	4.377	0.901	4.412	0.875	4.365	0.864
GLU-SER-THR	4.377	0.940	4.415	0.921	4.388	0.914
ALA-LYS-PHE	4.378	0.914	4.423	0.888	4.420	0.879
ARG-GLU-THR	4.381	0.933	4.397	0.913	4.353	0.905
ARG-GLU-SER	4.383	0.938	4.354	0.922	4.295	0.915
ASP-GLY-THR	4.383	0.935	4.492	0.911	4.487	0.902
ALA-HIS-LEU	4.387	0.858	4.377	0.833	4.340	0.831

ILE-LEU-PRO	4.388	0.912	4.391	0.888	4.338	0.881
ASP-ILE-THR	4.391	0.926	4.471	0.902	4.433	0.893
ARG-ILE-LYS	4.395	0.922	4.421	0.904	4.396	0.896
GLY-TYR-VAL	4.399	0.906	4.472	0.878	4.498	0.868
ALA-ASN-ILE	4.400	0.919	4.476	0.897	4.451	0.885
GLU-ILE-PHE	4.403	0.909	4.417	0.882	4.386	0.877
ASP-ILE-SER	4.404	0.928	4.447	0.908	4.393	0.901
ARG-ASP-GLU	4.404	0.934	4.370	0.913	4.321	0.906
LEU-PRO-THR	4.409	0.921	4.455	0.896	4.417	0.887
ASP-GLU-SER	4.412	0.940	4.406	0.920	4.366	0.914
GLN-ILE-VAL	4.412	0.912	4.461	0.888	4.429	0.881
GLU-TYR-VAL	4.414	0.905	4.403	0.877	4.373	0.868
ALA-ASN-GLU	4.414	0.932	4.442	0.910	4.420	0.897
GLN-GLU-VAL	4.419	0.924	4.397	0.900	4.351	0.892
ASP-LEU-PRO	4.420	0.917	4.428	0.890	4.366	0.884
ASP-PHE-VAL	4.422	0.911	4.472	0.880	4.453	0.876
ALA-GLU-TYR	4.430	0.911	4.443	0.885	4.410	0.872
ARG-PHE-VAL	4.431	0.906	4.451	0.878	4.447	0.877
GLU-GLY-PHE	4.435	0.920	4.469	0.893	4.484	0.885
ALA-PHE-THR	4.436	0.917	4.481	0.889	4.460	0.882
ARG-GLY-LYS	4.440	0.933	4.495	0.916	4.498	0.907
ASN-GLU-VAL	4.440	0.926	4.468	0.902	4.440	0.892
ARG-ILE-SER	4.441	0.926	4.448	0.909	4.404	0.904
ARG-GLU-PHE	4.444	0.911	4.446	0.885	4.424	0.881
ALA-ASP-PHE	4.457	0.915	4.473	0.886	4.451	0.879
GLN-LEU-PHE	4.459	0.904	4.485	0.875	4.441	0.868
ASP-GLU-THR	4.462	0.937	4.482	0.913	4.434	0.904
ARG-GLY-SER	4.463	0.938	4.495	0.922	4.450	0.917
GLU-PHE-SER	4.467	0.917	4.470	0.892	4.456	0.889
ASP-LYS-SER	4.472	0.935	4.497	0.917	4.485	0.908
LEU-PHE-TYR	4.474	0.891	4.463	0.860	4.405	0.851
ALA-ARG-PHE	4.476	0.912	4.471	0.887	4.447	0.882
GLN-GLU-ILE	4.490	0.919	4.494	0.896	4.445	0.886
ARG-LEU-PRO	4.491	0.917	4.492	0.895	4.454	0.889
GLU-PRO-VAL	4.493	0.916	4.464	0.889	4.448	0.884

Figure 10.1: Overlap between the significant FreSCOs found for the 1E-80, 1E-40 and 1E-7 protein sets.

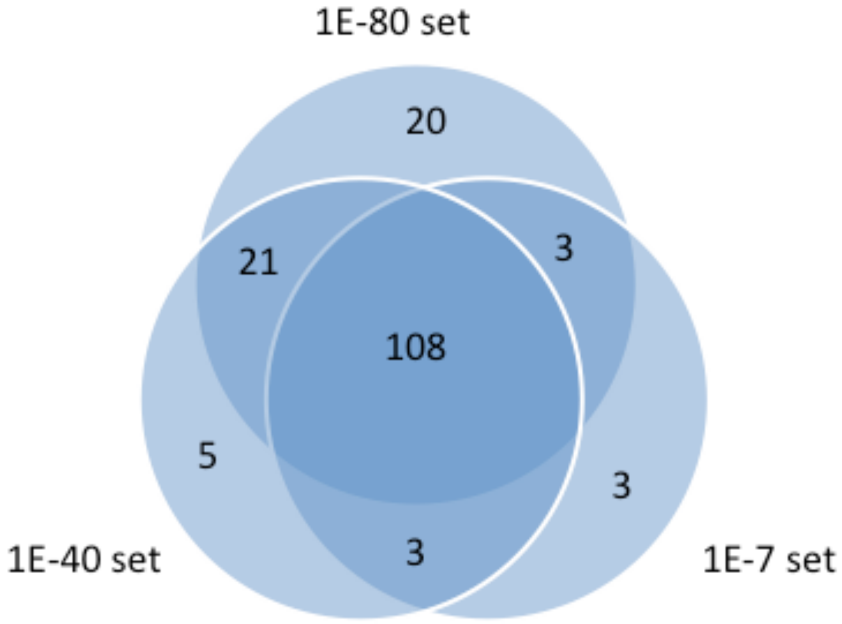

Supplement: Additional file 1: — Patterns mined from protein structure sets with lower redundancy. [file 13040_2015_38_MOESM1_ESM.pdf]
