# Supplementary material for: Mining the entire Protein DataBank for frequent spatially cohesive amino acid patterns
Source: BioData Min. 2015 Jan 31;8:4. doi: 10.1186/s13040-015-0038-4 (PMC4318390; doi:10.1186/s13040-015-0038-4)

Additional file 1: Smoothed kernel density of the cohesion radius of the FreSCO GLY-ILE-ARG from the permutated protein structures. One sample Kolmogorov-Smirnov test does not reject the null hypothesis that this data is derived from a normal distribution with a mean of 4.29 and a standard deviation of 0.0064 with a P-value of 0.9997.

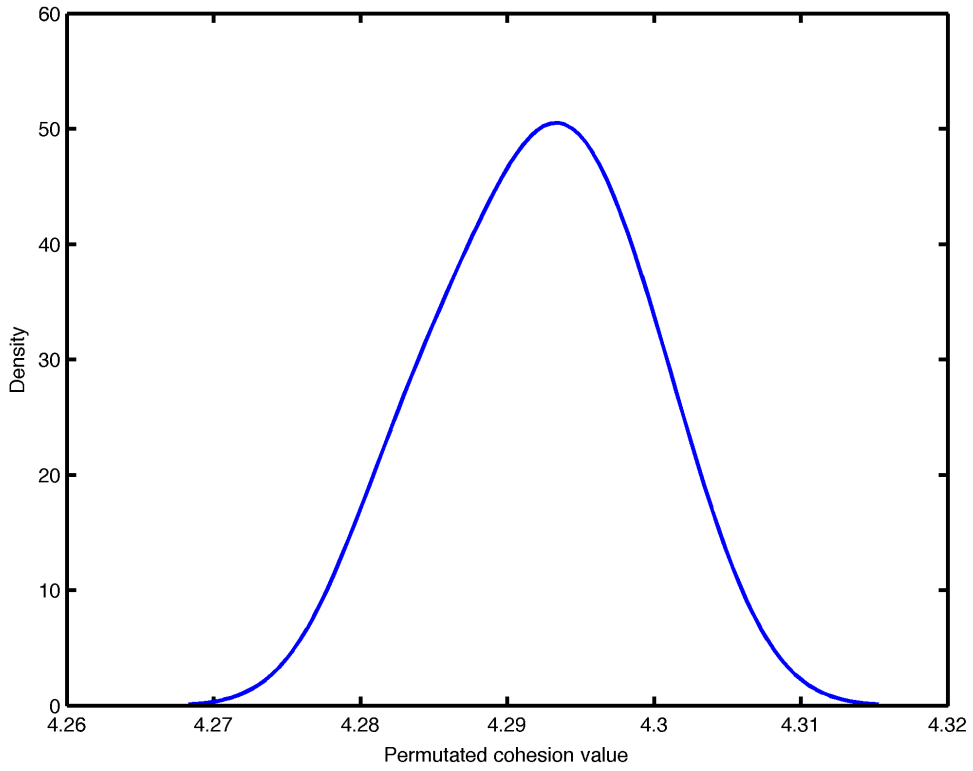

Supplement: Additional file 2: — Example of the cohesion radius distribution for permutated protein structures. [file 13040_2015_38_MOESM2_ESM.pdf]
