# Supplementary material for: Mining the entire Protein DataBank for frequent spatially cohesive amino acid patterns
Source: BioData Min. 2015 Jan 31;8:4. doi: 10.1186/s13040-015-0038-4 (PMC4318390; doi:10.1186/s13040-015-0038-4)
Supplement: Additional file 3: — Frequency and cohesion radius of the significant doublet and triplet FreSCOs in the non-redundant protein data set. [file 13040_2015_38_MOESM3_ESM.pdf]

Additional file 2: Frequency and cohesion radius of the significant doublet and triplet FreSCOs in the non-redundant protein data set. P-value based on the cohesion radius of the same patterns in permutated protein structures.

FreSCO	Frequency	Cohesion radius	P-value
PHE VAL ILE	0.90504	4.2507	<<1E-400
VAL TYR ILE	0.894866	4.3225	<<1E-400
ALA VAL ILE	0.932545	3.8108	<<1E-400
LEU ILE ARG	0.93407	3.8944	5.55E-315
PHE LEU ILE	0.914841	4.1614	2.12E-296
ALA PHE LEU	0.926478	3.9357	1.72E-280
TYR LEU ILE	0.90504	4.2218	3.89E-279
ALA LEU ILE	0.94496	3.6562	4.04E-239
ALA TYR ILE	0.900933	4.3768	2.71E-235
CYS VAL LEU	0.696329	4.4647	1.78E-227
THR LEU ILE	0.93715	3.9034	4.09E-223
VAL TYR LEU	0.909552	4.0297	8.85E-211
ALA TYR LEU	0.916086	4.0234	1.79E-208
ALA PHE VAL	0.915837	4.0853	2.16E-205
THR TYR LEU	0.91033	4.3649	3.32E-158
TYR TRP	0.712508	4.1223	1.51E-155
ALA PHE ILE	0.910361	4.2654	1.51E-154
PHE VAL LEU	0.920504	3.9803	8.17E-150
VAL LEU ARG	0.939235	3.8038	1.26E-144
GLU PHE LEU	0.925576	3.9872	7.49E-143
CYS ALA LEU	0.69972	4.4681	1.79E-139
LYS LEU ILE	0.937243	3.8368	1.36E-134
TYR CYS	0.687492	3.8926	2.94E-129
GLU LEU ILE	0.944151	3.7246	1.81E-121
VAL LEU ILE	0.937554	3.6816	7.30E-121
ILE CYS	0.694773	3.2757	3.23E-120
THR VAL ILE	0.927225	4.0483	5.52E-118
ALA VAL LEU	0.950404	3.5333	1.58E-117
PHE TYR	0.895395	3.1836	1.74E-116
ALA LEU ARG	0.94832	3.7968	2.69E-115
LYS TYR LEU	0.909303	4.2419	2.44E-113
TYR LEU ARG	0.907374	4.3991	1.71E-110
PHE VAL ARG	0.906129	4.4951	3.88E-104
PHE CYS	0.691101	3.5931	6.46E-100
ILE TYR	0.909956	2.8629	2.06E-97
VAL ILE ARG	0.92178	4.1	2.69E-95
HIS CYS	0.654636	4.4508	5.39E-95
ALA VAL TYR	0.905352	4.2085	2.98E-94
GLU ALA LEU	0.95753	3.5522	7.32E-92
ASN VAL ILE	0.914063	4.4034	1.54E-91

ILE PHE	0.919104	2.7912	7.56E-88
ALA THR ILE	0.932172	4.1303	1.53E-87
PHE LEU ARG	0.916801	4.2971	6.48E-86
ALA ILE ARG	0.929309	4.0988	1.78E-83
LYS VAL LEU	0.942937	3.6753	3.81E-83
VAL CYS	0.701711	2.9799	1.89E-80
PHE TRP	0.718575	3.918	2.44E-80
ASN LEU ILE	0.923802	4.2024	6.29E-79
ALA CYS	0.7056	3.0052	2.00E-77
LEU GLN ILE	0.923211	4.1465	4.84E-77
LEU CYS	0.707996	2.7738	1.58E-76
ASP VAL ILE	0.926727	4.0825	3.53E-73
ALA LYS ILE	0.932576	4.0242	3.18E-72
ALA LYS LEU	0.951213	3.6599	6.35E-71
GLU PHE ILE	0.90949	4.4034	1.41E-65
GLU ILE ARG	0.927878	4.1368	1.00E-62
ARG TYR	0.913441	2.9867	1.24E-62
THR ILE ARG	0.922246	4.4326	1.18E-59
LYS VAL ILE	0.925762	4.0136	3.88E-58
GLU LEU ARG	0.946702	3.7695	1.37E-57
PHE THR VAL	0.911761	4.4123	3.80E-57
LEU TRP	0.734319	2.9795	2.69E-55
PHE THR LEU	0.921157	4.3154	5.54E-53
THR VAL LEU	0.942937	3.7865	1.21E-52
GLU LYS LEU	0.95028	3.6629	7.26E-52
GLU PHE VAL	0.91509	4.1815	1.73E-49
ASP ILE ARG	0.922309	4.358	2.41E-48
ASN ALA ILE	0.919353	4.4045	9.05E-48
GLU VAL LEU	0.949253	3.5921	1.13E-47
THR CYS	0.704574	3.3882	1.59E-46
ALA LEU GLN	0.935594	3.9914	8.91E-46
GLN TRP	0.718544	4.1039	1.77E-45
VAL TRP	0.728531	3.2336	4.76E-45
VAL PHE	0.924798	2.5831	4.37E-44
ILE TRP	0.724362	3.5272	4.81E-44
ASP LEU ILE	0.936777	3.9315	2.00E-42
ALA THR LEU	0.949035	3.8111	2.58E-41
GLU PHE ARG	0.911325	4.4443	2.12E-40
GLU VAL ILE	0.930989	3.934	4.90E-38
VAL GLN ILE	0.91229	4.4123	1.77E-36
ALA GLN ILE	0.918606	4.3621	9.42E-36
GLU LYS ILE	0.931301	4.0574	1.64E-35
VAL LEU GLN	0.928158	4.053	1.83E-35
PHE LYS VAL	0.90921	4.4124	6.17E-33
PHE HIS	0.840386	3.6749	1.15E-31

ALA LEU HIS	0.85809	4.4068	1.59E-30
ALA VAL ARG	0.934536	4.001	2.94E-30
ASP VAL LEU	0.942097	3.7847	1.24E-29
ILE THR	0.942844	2.574	1.27E-29
ASN ALA LEU	0.936341	4.0355	1.56E-29
LEU PHE	0.936279	2.4709	7.31E-29
TYR HIS	0.832327	3.8447	1.78E-28
ASP TYR LEU	0.910143	4.36	4.71E-28
GLU TYR LEU	0.916273	4.1405	6.35E-28
LYS VAL TYR	0.899347	4.4709	1.13E-26
VAL LEU HIS	0.854045	4.4209	3.19E-26
ASN VAL LEU	0.930274	4.0623	4.11E-26
ILE ARG	0.939546	2.5732	8.00E-24
LEU GLN ARG	0.925109	4.2862	8.18E-24
GLU ALA PHE	0.920473	4.2072	5.19E-23
GLU ALA ILE	0.938177	3.908	3.21E-22
THR PHE	0.926042	2.862	5.20E-22
GLU ALA VAL	0.943746	3.7623	8.08E-22
GLU LYS VAL	0.937337	3.8919	3.71E-20
ALA PHE ARG	0.912352	4.4759	1.14E-19
LEU TYR	0.926011	2.5307	1.28E-19
ALA PHE THR	0.917082	4.4363	1.44E-19
GLU PHE LYS	0.913752	4.3765	1.67E-19
GLU ALA LYS	0.944742	3.8518	3.38E-19
ARG TRP	0.726416	3.6344	1.03E-18
PHE LYS LEU	0.918762	4.2395	1.87E-18
GLY VAL TYR	0.906067	4.3988	3.34E-18
ILE MET	0.704107	3.3326	7.07E-18
GLU VAL ARG	0.933261	4.0128	5.50E-17
GLU ALA ARG	0.941381	4.0144	2.71E-16
VAL TYR	0.914312	2.6893	8.51E-15
ALA PHE SER	0.918046	4.3527	2.37E-14
VAL ILE	0.942533	2.4042	2.89E-13
ALA THR VAL	0.938519	3.9917	9.45E-13
PHE LEU SER	0.922744	4.2451	1.13E-12
PHE MET	0.691973	3.8125	3.17E-12
LEU GLN	0.946329	2.4662	3.59E-12
TYR LEU SER	0.912539	4.3759	3.78E-12
PHE GLN	0.908152	3.164	4.05E-12
ALA PHE LYS	0.91397	4.3778	4.25E-12
ILE GLN	0.928127	2.8328	1.20E-11
ARG PHE	0.92178	2.8829	1.69E-11
LEU HIS	0.866615	2.742	3.65E-10
LYS LEU GLN	0.928065	4.191	5.30E-10
ALA VAL GLN	0.924144	4.2532	6.16E-10

ILE HIS	0.849938	3.22	8.67E-10
LEU SER ILE	0.940292	3.9085	1.60E-09
LYS TRP	0.725762	3.531	3.39E-09
ALA LYS VAL	0.938488	3.8878	9.09E-09
GLY TYR LEU	0.915121	4.245	4.29E-08
THR TYR	0.915619	3.0041	6.87E-08
PHE VAL SER	0.913037	4.4338	1.30E-07
ALA ASP ILE	0.932234	4.1305	1.84E-07
ASP LEU ARG	0.938519	4.0262	2.46E-07
GLU LEU GLN	0.935874	4.0806	5.74E-07
ALA TYR	0.921717	2.757	2.15E-06
TYR MET	0.681269	4.0557	4.24E-06
THR LEU GLN	0.926198	4.3498	6.40E-06
ASN PHE	0.912321	3.1703	8.27E-06
ALA PHE	0.931269	2.6472	9.19E-06
ASN TRP	0.719073	4.0865	1.21E-05
GLU VAL TYR	0.905227	4.3791	1.21E-05
GLN TYR	0.900809	3.348	4.28E-05
PHE ASP VAL	0.910797	4.4892	6.65E-05
LEU ALA	0.971935	2.206	0.000106349
LEU ARG	0.960019	2.3368	0.000140656
ARG CYS	0.700778	3.5344	0.000166226
