# Supplementary material for: Mining the entire Protein DataBank for frequent spatially cohesive amino acid patterns
Source: BioData Min. 2015 Jan 31;8:4. doi: 10.1186/s13040-015-0038-4 (PMC4318390; doi:10.1186/s13040-015-0038-4)
Supplement: Additional file 4: — Domain enrichment and depletion P-values for each mined FreSCO. [file 13040_2015_38_MOESM4_ESM.pdf]

Additional file 3: Domain enrichment and depletion P-values for each mined FreSCO. Significance calculated based on a hypergeometric distribution with a P-value cut-off of 4.808E-5.

FreSCO	Enrichment P-value	Depletion P-value
CYS VAL LEU	1.456E-71	1.000
CYS ALA LEU	3.349E-70	1.000
VAL TYR ILE	4.026E-60	1.000
ALA TYR ILE	3.260E-48	1.000
ALA PHE ILE	5.425E-46	1.000
ALA VAL ILE	1.497E-44	1.000
GLY VAL TYR	1.433E-43	1.000
TYR LEU ILE	1.552E-43	1.000
PHE VAL ILE	1.858E-43	1.000
VAL TYR LEU	1.199E-42	1.000
GLY TYR LEU	4.348E-39	1.000
VAL LEU ILE	2.151E-38	1.000
PHE LEU ILE	3.558E-36	1.000
ALA PHE VAL	2.230E-33	1.000
ALA VAL TYR	1.848E-30	1.000
ALA LEU ILE	2.290E-29	1.000
ALA THR ILE	2.679E-29	1.000
ALA PHE THR	1.352E-28	1.000
ASP VAL ILE	1.736E-28	1.000
ALA PHE LEU	1.175E-27	1.000
THR VAL ILE	6.450E-27	1.000
ALA TYR LEU	8.671E-26	1.000
PHE THR VAL	3.854E-22	1.000
PHE VAL LEU	8.093E-22	1.000
ASN VAL ILE	1.250E-21	1.000
ALA VAL LEU	4.737E-21	1.000
PHE THR LEU	1.334E-20	1.000
ASP VAL LEU	2.326E-20	1.000
PHE ASP VAL	2.947E-20	1.000
ASN ALA ILE	1.792E-19	1.000
ALA ILE ARG	9.967E-19	1.000
ALA THR VAL	6.566E-18	1.000
VAL ILE ARG	9.530E-18	1.000
VAL LEU HIS	3.345E-17	1.000
ALA PHE ARG	1.432E-16	1.000
ASP TYR LEU	3.529E-16	1.000
PHE VAL ARG	8.777E-16	1.000
PHE VAL SER	3.273E-15	1.000
THR TYR LEU	5.312E-14	1.000

ASP LEU ILE	7.401E-14	1.000
ALA ASP ILE	2.397E-11	1.000
ASN VAL LEU	4.205E-11	1.000
ALA LEU HIS	1.690E-10	1.000
ALA VAL ARG	2.237E-10	1.000
THR LEU ILE	9.919E-10	1.000
VAL GLN ILE	1.321E-09	1.000
GLU VAL TYR	8.304E-09	1.000
ASN LEU ILE	1.834E-08	1.000
GLU ALA VAL	2.862E-08	1.000
ALA THR LEU	3.717E-07	1.000
ASN ALA LEU	6.766E-07	1.000
GLU ALA PHE	1.220E-06	1.000
PHE LEU ARG	1.708E-06	1.000
TYR LEU ARG	3.595E-06	1.000
ALA LYS VAL	7.051E-06	1.000
TYR LEU SER	7.425E-06	1.000
LEU SER ILE	9.867E-06	1.000
ALA PHE LYS	1.331E-05	1.000
ALA PHE SER	2.425E-05	1.000
ALA GLN ILE	2.567E-05	1.000
VAL LEU ARG	2.643E-05	1.000
PHE LEU SER	6.728E-05	1.000
ASP ILE ARG	7.025E-05	1.000
PHE LYS LEU	1.237E-04	1.000
GLU ALA ILE	2.299E-04	1.000
ALA VAL GLN	3.150E-04	1.000
THR ILE ARG	3.421E-04	1.000
LYS VAL ILE	3.631E-04	1.000
GLU PHE VAL	8.073E-04	0.999
GLU PHE ILE	8.544E-04	0.999
LEU ILE ARG	8.658E-04	0.999
PHE LYS VAL	2.338E-03	0.998
GLU VAL ILE	4.108E-03	0.996
ALA LYS ILE	4.935E-03	0.995
GLU TYR LEU	5.488E-03	0.995
LEU GLN ILE	6.211E-03	0.994
ALA LEU ARG	7.759E-03	0.992
THR VAL LEU	8.367E-03	0.992
GLU VAL ARG	0.012	0.988
GLU VAL LEU	0.019	0.982
LYS VAL TYR	0.028	0.973
VAL LEU GLN	0.045	0.956
GLU PHE ARG	0.049	0.952
ALA LEU GLN	0.067	0.934

GLU PHE LEU	0.098	0.904
LYS VAL LEU	0.189	0.814
ASP LEU ARG	0.329	0.675
GLU ALA LYS	0.437	0.567
LYS LEU ILE	0.475	0.529
ALA LYS LEU	0.580	0.424
GLU LYS VAL	0.624	0.380
GLU ALA ARG	0.675	0.328
GLU ALA LEU	0.734	0.269
GLU ILE ARG	0.922	0.079
LEU GLN ARG	0.939	0.062
LYS TYR LEU	0.956	0.045
GLU PHE LYS	1.000	1.971E-04
THR LEU GLN	1.000	3.154E-05
GLU LEU ARG	1.000	3.059E-05
GLU LEU ILE	1.000	1.111E-05
LYS LEU GLN	1.000	5.172E-06
GLU LEU GLN	1.000	4.895E-11
GLU LYS ILE	1.000	2.052E-12
GLU LYS LEU	1.000	3.210E-16
