# Supplementary material for: Mining the entire Protein DataBank for frequent spatially cohesive amino acid patterns
Source: BioData Min. 2015 Jan 31;8:4. doi: 10.1186/s13040-015-0038-4 (PMC4318390; doi:10.1186/s13040-015-0038-4)
Supplement: Additional file 5: — Domain enrichment and depletion P-values of individual amino acids. [file 13040_2015_38_MOESM5_ESM.pdf]

Additional file 4: Domain enrichment and depletion P-values of individual amino acids. Significance calculated based on a hypergeometric distribution with a P-value cut-off of 2.27E-4.

Amino acid	Fraction in conserved domains	Enrichment P-value	Depletion P-value
ASX	1	0.596	1.000
CYS	0.836	<<1E-200	1.000
VAL	0.79	2.67E-191	1.000
ILE	0.789	9.23E-134	1.000
TYR	0.786	8.03E-56	1.000
TRP	0.786	1.39E-21	1.000
GLY	0.786	7.20E-112	1.000
ASN	0.781	3.42E-30	1.000
PHE	0.781	5.76E-27	1.000
ALA	0.78	6.13E-47	1.000
THR	0.779	6.14E-23	1.000
ASP	0.777	4.94E-15	1.000
LEU	0.771	0.896	0.104
GLN	0.767	1.000	3.60E-07
LYS	0.768	1.000	1.73E-08
HIS	0.766	1.000	6.45E-08
MET	0.765	1.000	7.81E-08
ARG	0.765	1.000	1.85E-18
GLU	0.762	1.000	1.84E-51
SER	0.744	1.000	<<1E-200
PRO	0.737	1.000	<<1E-200
XAA	0.474	1.000	<<1E-200
