# Supplementary material for: Mining the entire Protein DataBank for frequent spatially cohesive amino acid patterns
Source: BioData Min. 2015 Jan 31;8:4. doi: 10.1186/s13040-015-0038-4 (PMC4318390; doi:10.1186/s13040-015-0038-4)
Supplement: Additional file 6: — Gene ontology enrichment for the 104 significant FreSCOs. [file 13040_2015_38_MOESM6_ESM.pdf]

Additional file 8: Gene ontology enrichment for the 104 significant FreSCOs. Only FreSCOs enriched with a P-value lower than a significance cut-off adjusted for multiple testing of 1.25E-7 (0.01/104 FreSCOs and 770 GO terms) are reported.

Ontology	Gene Ontology term	FreSCO	P-value
molecular function	nucleotide binding	VAL LEU GLN	1.82E-09
molecular function	nucleotide binding	LYS VAL LEU	2.90E-13
molecular function	nucleotide binding	ALA LEU ARG	5.22E-15
molecular function	nucleotide binding	LYS LEU ILE	5.52E-13
molecular function	nucleotide binding	ALA VAL ILE	4.13E-12
molecular function	nucleotide binding	PHE VAL SER	9.00E-10
molecular function	nucleotide binding	ASP VAL LEU	1.73E-16
molecular function	nucleotide binding	GLU LEU GLN	1.22E-10
molecular function	nucleotide binding	ALA PHE ILE	4.30E-11
molecular function	nucleotide binding	ALA PHE LEU	6.07E-08
molecular function	nucleotide binding	GLU ALA ILE	1.67E-21
molecular function	nucleotide binding	ASN LEU ILE	2.16E-13
molecular function	nucleotide binding	GLU ALA LYS	1.53E-11
molecular function	nucleotide binding	TYR LEU ARG	1.07E-08
molecular function	nucleotide binding	LEU ILE ARG	3.36E-10
molecular function	nucleotide binding	ASP VAL ILE	1.42E-08
molecular function	nucleotide binding	ALA ILE ARG	1.10E-12
molecular function	nucleotide binding	ALA PHE THR	2.52E-08
molecular function	nucleotide binding	LYS LEU GLN	3.46E-10
molecular function	nucleotide binding	THR VAL ILE	6.75E-14
molecular function	nucleotide binding	LYS VAL ILE	6.45E-15
molecular function	nucleotide binding	PHE VAL ILE	1.56E-15
molecular function	nucleotide binding	VAL LEU ILE	2.62E-25
molecular function	nucleotide binding	ASP LEU ARG	3.19E-16
molecular function	nucleotide binding	ASP LEU ILE	3.03E-09
molecular function	nucleotide binding	ASN VAL LEU	4.37E-11
molecular function	nucleotide binding	VAL LEU ARG	9.02E-12
molecular function	nucleotide binding	GLU ALA LEU	2.53E-18
molecular function	nucleotide binding	ASP ILE ARG	8.23E-13
molecular function	nucleotide binding	VAL GLN ILE	1.21E-09
molecular function	nucleotide binding	ALA LYS ILE	1.59E-17
molecular function	nucleotide binding	ALA TYR LEU	1.71E-10
molecular function	nucleotide binding	ALA GLN ILE	7.50E-13
molecular function	nucleotide binding	GLU VAL ARG	2.85E-17
molecular function	nucleotide binding	GLY VAL TYR	1.96E-08
molecular function	nucleotide binding	GLU LYS VAL	2.59E-13
molecular function	nucleotide binding	GLU PHE LYS	1.98E-11
molecular function	nucleotide binding	ALA LYS LEU	2.63E-19
molecular function	nucleotide binding	GLU ALA VAL	9.25E-15

molecular function	nucleotide binding	GLU PHE ARG	8.68E-09
molecular function	nucleotide binding	ASN ALA LEU	2.90E-11
molecular function	nucleotide binding	THR ILE ARG	3.77E-13
molecular function	nucleotide binding	PHE VAL LEU	4.50E-09
molecular function	nucleotide binding	ALA THR LEU	7.73E-10
molecular function	nucleotide binding	THR VAL LEU	1.96E-09
molecular function	nucleotide binding	GLU VAL ILE	4.54E-17
molecular function	nucleotide binding	GLU LEU ARG	1.00E-21
molecular function	nucleotide binding	ALA THR VAL	3.35E-15
molecular function	nucleotide binding	LEU GLN ILE	1.04E-09
molecular function	nucleotide binding	GLU LYS ILE	8.68E-14
molecular function	nucleotide binding	LEU SER ILE	2.44E-11
molecular function	nucleotide binding	PHE LEU ILE	1.24E-08
molecular function	nucleotide binding	GLY TYR LEU	3.03E-10
molecular function	nucleotide binding	ASN ALA ILE	1.67E-12
molecular function	nucleotide binding	PHE ASP VAL	1.78E-08
molecular function	nucleotide binding	GLU PHE LEU	1.91E-09
molecular function	nucleotide binding	ALA TYR ILE	3.20E-09
molecular function	nucleotide binding	GLU LYS LEU	7.14E-20
molecular function	nucleotide binding	GLU ILE ARG	1.80E-19
molecular function	nucleotide binding	ALA VAL LEU	6.26E-13
molecular function	nucleotide binding	THR LEU GLN	2.30E-11
molecular function	nucleotide binding	ALA THR ILE	2.48E-14
molecular function	nucleotide binding	ALA PHE VAL	5.11E-08
molecular function	nucleotide binding	ALA LYS VAL	1.73E-09
molecular function	nucleotide binding	ALA LEU GLN	2.02E-12
molecular function	nucleotide binding	ASN VAL ILE	4.05E-09
molecular function	nucleotide binding	ALA VAL ARG	6.15E-13
molecular function	nucleotide binding	GLU ALA ARG	1.77E-22
molecular function	nucleotide binding	GLU PHE ILE	3.22E-08
molecular function	nucleotide binding	VAL ILE ARG	4.42E-11
molecular function	nucleotide binding	GLU VAL LEU	3.80E-20
molecular function	nucleotide binding	ALA LEU ILE	9.47E-13
molecular function	nucleotide binding	GLU ALA PHE	5.28E-11
molecular function	nucleotide binding	ALA VAL GLN	3.83E-08
molecular function	nucleotide binding	ALA ASP ILE	1.99E-09
molecular function	nucleotide binding	LEU GLN ARG	2.59E-09
molecular function	nucleotide binding	GLU LEU ILE	5.09E-20
molecular function	lyase activity	ALA VAL ARG	1.04E-07
molecular function	lyase activity	GLU ALA ILE	3.13E-10
molecular function	lyase activity	GLU ALA ARG	4.28E-12
molecular function	lyase activity	ALA LEU ARG	2.48E-08
molecular function	lyase activity	ASN ALA LEU	1.70E-08
molecular function	lyase activity	ALA LEU ILE	8.16E-11
molecular function	lyase activity	ALA THR LEU	6.61E-09

molecular function	lyase activity	ALA VAL ILE	4.23E-08
molecular function	lyase activity	ALA PHE LEU	1.07E-08
molecular function	magnesium ion binding	ALA ASP ILE	6.88E-08
molecular function	magnesium ion binding	GLU VAL LEU	5.46E-08
molecular function	DNA binding	LEU ILE ARG	8.83E-08
molecular function	transferase activity	LYS VAL LEU	2.54E-09
molecular function	transferase activity	ALA LEU ARG	5.98E-21
molecular function	transferase activity	LYS LEU ILE	5.38E-09
molecular function	transferase activity	ALA VAL ILE	5.42E-13
molecular function	transferase activity	ASP VAL LEU	1.34E-15
molecular function	transferase activity	GLU LEU GLN	7.12E-11
molecular function	transferase activity	ALA PHE ILE	1.33E-10
molecular function	transferase activity	ALA PHE ARG	2.18E-14
molecular function	transferase activity	ALA PHE LEU	3.22E-10
molecular function	transferase activity	GLU ALA ILE	1.19E-25
molecular function	transferase activity	ASN LEU ILE	3.93E-13
molecular function	transferase activity	ASP TYR LEU	3.39E-10
molecular function	transferase activity	GLU ALA LYS	1.43E-09
molecular function	transferase activity	TYR LEU ARG	8.29E-09
molecular function	transferase activity	GLU VAL TYR	4.02E-08
molecular function	transferase activity	LEU ILE ARG	1.52E-17
molecular function	transferase activity	ASP VAL ILE	4.57E-11
molecular function	transferase activity	ALA ILE ARG	9.77E-19
molecular function	transferase activity	LYS VAL ILE	3.70E-08
molecular function	transferase activity	VAL LEU ILE	3.67E-15
molecular function	transferase activity	ASP LEU ARG	1.35E-20
molecular function	transferase activity	ASP LEU ILE	8.42E-12
molecular function	transferase activity	VAL LEU ARG	7.72E-10
molecular function	transferase activity	GLU ALA LEU	1.92E-21
molecular function	transferase activity	ASP ILE ARG	6.87E-13
molecular function	transferase activity	ALA VAL TYR	1.13E-08
molecular function	transferase activity	ALA TYR LEU	3.21E-11
molecular function	transferase activity	GLU VAL ARG	3.45E-18
molecular function	transferase activity	GLU LYS VAL	6.31E-12
molecular function	transferase activity	GLU PHE LYS	2.07E-08
molecular function	transferase activity	ALA LYS LEU	3.72E-11
molecular function	transferase activity	GLU ALA VAL	5.81E-16
molecular function	transferase activity	GLU PHE ARG	5.24E-15
molecular function	transferase activity	ASN ALA LEU	6.53E-10
molecular function	transferase activity	ALA THR LEU	2.88E-09
molecular function	transferase activity	GLU VAL ILE	5.03E-16
molecular function	transferase activity	GLU LEU ARG	1.03E-21
molecular function	transferase activity	ALA LEU HIS	1.01E-08
molecular function	transferase activity	LEU GLN ILE	1.20E-09
molecular function	transferase activity	GLU LYS ILE	2.58E-12

molecular function	transferase activity	LEU SER ILE	8.85E-14
molecular function	transferase activity	GLY TYR LEU	6.79E-10
molecular function	transferase activity	GLU PHE LEU	2.16E-13
molecular function	transferase activity	GLU LYS LEU	2.25E-21
molecular function	transferase activity	GLU TYR LEU	2.81E-21
molecular function	transferase activity	ALA TYR ILE	4.11E-09
molecular function	transferase activity	GLU ILE ARG	2.25E-15
molecular function	transferase activity	ALA VAL LEU	8.62E-20
molecular function	transferase activity	TYR LEU SER	1.77E-11
molecular function	transferase activity	ALA THR ILE	2.67E-16
molecular function	transferase activity	ALA PHE VAL	1.05E-09
molecular function	transferase activity	ALA LYS VAL	7.18E-09
molecular function	transferase activity	ALA LEU GLN	7.34E-11
molecular function	transferase activity	TYR LEU ILE	7.88E-12
molecular function	transferase activity	PHE VAL ARG	4.01E-10
molecular function	transferase activity	ALA VAL ARG	1.44E-18
molecular function	transferase activity	GLU ALA ARG	1.84E-22
molecular function	transferase activity	GLU VAL LEU	1.51E-24
molecular function	transferase activity	ALA LEU ILE	9.52E-13
molecular function	transferase activity	VAL ILE ARG	3.03E-15
molecular function	transferase activity	GLU ALA PHE	3.14E-18
molecular function	transferase activity	ALA ASP ILE	6.15E-14
molecular function	transferase activity	LEU GLN ARG	4.31E-11
molecular function	transferase activity	GLU LEU ILE	1.45E-30
molecular function	phosphoprotein binding	PHE VAL SER	1.52E-09
molecular function	insulin receptor binding	CYS VAL LEU	9.22E-11
molecular function	oxygen binding	ALA LEU HIS	2.48E-08
molecular function	oxygen binding	PHE LYS LEU	1.99E-09
molecular function	catalytic activity	ALA LYS LEU	1.48E-11
molecular function	catalytic activity	CYS ALA LEU	4.97E-11
molecular function	catalytic activity	ALA LEU ARG	1.29E-18
molecular function	catalytic activity	ASN ALA LEU	9.49E-12
molecular function	catalytic activity	ALA THR LEU	2.29E-11
molecular function	catalytic activity	GLU LEU ARG	1.96E-19
molecular function	catalytic activity	VAL LEU ARG	9.66E-10
molecular function	catalytic activity	ALA VAL ARG	4.98E-29
molecular function	catalytic activity	GLU ALA ARG	1.50E-16
molecular function	catalytic activity	GLU ALA LYS	8.84E-18
molecular function	catalytic activity	GLU ALA LEU	1.80E-25
molecular function	catalytic activity	ALA LEU ILE	1.46E-08
molecular function	catalytic activity	ASN ALA ILE	5.19E-08
molecular function	catalytic activity	ALA LYS ILE	1.12E-08
molecular function	catalytic activity	ALA ASP ILE	1.92E-11
molecular function	catalytic activity	ALA ILE ARG	1.91E-17
molecular function	catalytic activity	GLU VAL ARG	2.35E-09

molecular function	ATP binding	ALA LYS LEU	2.63E-12
molecular function	ATP binding	GLU PHE LYS	1.93E-08
molecular function	ATP binding	GLU PHE ARG	2.76E-08
molecular function	ATP binding	GLU ALA VAL	1.68E-09
molecular function	ATP binding	LYS VAL LEU	1.12E-08
molecular function	ATP binding	ALA LEU ARG	1.55E-13
molecular function	ATP binding	THR ILE ARG	2.61E-12
molecular function	ATP binding	LYS LEU ILE	2.96E-10
molecular function	ATP binding	GLU VAL ILE	5.52E-16
molecular function	ATP binding	PHE VAL SER	5.30E-08
molecular function	ATP binding	ASP VAL LEU	5.70E-08
molecular function	ATP binding	GLU LEU GLN	1.25E-10
molecular function	ATP binding	GLU LEU ARG	1.26E-15
molecular function	ATP binding	GLU ALA ILE	2.12E-17
molecular function	ATP binding	LEU GLN ILE	2.06E-16
molecular function	ATP binding	GLU LYS ILE	6.92E-12
molecular function	ATP binding	ASN LEU ILE	6.33E-10
molecular function	ATP binding	LEU SER ILE	2.00E-13
molecular function	ATP binding	TYR LEU ARG	7.41E-08
molecular function	ATP binding	LEU ILE ARG	7.63E-09
molecular function	ATP binding	PHE ASP VAL	2.42E-08
molecular function	ATP binding	ASN ALA ILE	7.07E-08
molecular function	ATP binding	ASP VAL ILE	6.63E-08
molecular function	ATP binding	GLU LYS LEU	7.36E-12
molecular function	ATP binding	ALA ILE ARG	2.37E-12
molecular function	ATP binding	GLU ILE ARG	7.17E-24
molecular function	ATP binding	ALA VAL LEU	6.52E-08
molecular function	ATP binding	THR LEU GLN	1.28E-10
molecular function	ATP binding	ALA THR ILE	1.40E-09
molecular function	ATP binding	LYS VAL ILE	2.65E-15
molecular function	ATP binding	PHE VAL ILE	6.80E-11
molecular function	ATP binding	VAL LEU ILE	1.62E-14
molecular function	ATP binding	ASP LEU ARG	3.45E-12
molecular function	ATP binding	ASP LEU ILE	4.09E-10
molecular function	ATP binding	ALA LEU GLN	4.72E-11
molecular function	ATP binding	ASN VAL LEU	1.11E-09
molecular function	ATP binding	VAL LEU ARG	4.16E-09
molecular function	ATP binding	ALA VAL ARG	1.11E-08
molecular function	ATP binding	PHE VAL ARG	7.96E-08
molecular function	ATP binding	GLU ALA ARG	2.71E-19
molecular function	ATP binding	GLU ALA LEU	3.05E-11
molecular function	ATP binding	ASP ILE ARG	2.25E-12
molecular function	ATP binding	VAL ILE ARG	3.02E-12
molecular function	ATP binding	VAL GLN ILE	2.28E-09
molecular function	ATP binding	GLU VAL LEU	1.21E-18

molecular function	ATP binding	ALA LYS ILE	2.46E-08
molecular function	ATP binding	LEU GLN ARG	1.30E-08
molecular function	ATP binding	ALA GLN ILE	3.92E-10
molecular function	ATP binding	GLU VAL ARG	4.16E-16
molecular function	ATP binding	GLU LEU ILE	1.86E-19
molecular function	ATP binding	GLU LYS VAL	1.47E-08
molecular function	insulin-like growth factor receptor binding	ALA TYR LEU	7.35E-08
molecular function	insulin-like growth factor receptor binding	CYS VAL LEU	1.87E-11
molecular function	nucleoside diphosphate kinase activity	ASP ILE ARG	1.27E-09
molecular function	nucleoside diphosphate kinase activity	THR ILE ARG	5.90E-09
molecular function	ephrin receptor binding	PHE LEU SER	3.66E-08
molecular function	oxidoreductase activity	ALA LYS LEU	1.05E-10
molecular function	oxidoreductase activity	GLU ALA VAL	2.93E-12
molecular function	oxidoreductase activity	ALA LEU ARG	4.71E-11
molecular function	oxidoreductase activity	ALA VAL ILE	1.76E-12
molecular function	oxidoreductase activity	THR VAL LEU	2.70E-16
molecular function	oxidoreductase activity	ALA THR LEU	2.57E-13
molecular function	oxidoreductase activity	PHE VAL SER	6.01E-08
molecular function	oxidoreductase activity	ASP VAL LEU	6.80E-08
molecular function	oxidoreductase activity	GLU LEU ARG	1.48E-08
molecular function	oxidoreductase activity	ALA PHE ILE	3.12E-08
molecular function	oxidoreductase activity	ALA LEU HIS	2.66E-10
molecular function	oxidoreductase activity	ALA PHE LEU	1.81E-13
molecular function	oxidoreductase activity	ALA PHE SER	5.75E-12
molecular function	oxidoreductase activity	GLU ALA ILE	2.26E-14
molecular function	oxidoreductase activity	ALA THR VAL	7.26E-14
molecular function	oxidoreductase activity	GLY TYR LEU	1.69E-10
molecular function	oxidoreductase activity	ALA VAL LEU	1.85E-13
molecular function	oxidoreductase activity	ALA PHE THR	6.33E-15
molecular function	oxidoreductase activity	ALA PHE LYS	8.65E-13
molecular function	oxidoreductase activity	ALA THR ILE	4.28E-09
molecular function	oxidoreductase activity	THR VAL ILE	1.09E-07
molecular function	oxidoreductase activity	VAL LEU HIS	9.96E-09
molecular function	oxidoreductase activity	VAL LEU ILE	5.31E-13
molecular function	oxidoreductase activity	ASP LEU ARG	1.63E-08
molecular function	oxidoreductase activity	ALA LEU GLN	7.16E-08
molecular function	oxidoreductase activity	ALA VAL ARG	5.13E-11
molecular function	oxidoreductase activity	GLU ALA ARG	3.59E-09
molecular function	oxidoreductase activity	GLU ALA LEU	3.39E-16
molecular function	oxidoreductase activity	ALA VAL TYR	7.56E-13
molecular function	oxidoreductase activity	ALA LEU ILE	2.14E-15

molecular function	oxidoreductase activity	ALA TYR LEU	3.82E-08
molecular function	oxidoreductase activity	GLU ALA PHE	2.66E-19
molecular function	oxidoreductase activity	ALA ASP ILE	1.39E-08
molecular function	oxidoreductase activity	GLY VAL TYR	8.35E-08
molecular function	lysozyme activity	ASN ALA ILE	1.09E-08
molecular function	lysozyme activity	ALA VAL ARG	2.26E-13
molecular function	lysozyme activity	ALA ILE ARG	4.57E-12
molecular function	lysozyme activity	GLU LEU ARG	7.55E-10
molecular function	lysozyme activity	CYS ALA LEU	1.39E-29
molecular function	lysozyme activity	GLU ALA LYS	7.00E-12
molecular function	transaminase activity	ALA LYS LEU	1.08E-08
molecular function	transaminase activity	GLU ALA ILE	3.52E-08
molecular function	transaminase activity	GLU LYS LEU	2.68E-08
molecular function	oxygen transporter activity	ALA THR VAL	9.89E-10
molecular function	oxygen transporter activity	ALA LEU HIS	6.31E-11
molecular function	oxygen transporter activity	ALA PHE LYS	2.61E-09
molecular function	oxygen transporter activity	PHE LYS LEU	4.24E-16
molecular function	oxygen transporter activity	PHE LYS VAL	1.69E-10
molecular function	protease binding	CYS VAL LEU	7.80E-10
molecular function	hormone binding	GLU PHE ARG	6.93E-09
molecular function	hormone binding	ALA PHE VAL	1.08E-07
molecular function	hormone binding	ASN VAL ILE	3.53E-10
molecular function	hormone binding	PHE LYS VAL	1.57E-10
molecular function	ligase activity	ALA LEU ARG	3.33E-08
molecular function	ligase activity	TYR LEU ARG	4.08E-08
molecular function	hydrolase activity, acting on glycosyl bonds	ALA VAL ARG	3.08E-11
molecular function	hydrolase activity, acting on glycosyl bonds	ALA ILE ARG	7.75E-10
molecular function	hydrolase activity, acting on glycosyl bonds	CYS ALA LEU	1.29E-09
molecular function	hydrolase activity, acting on glycosyl bonds	GLU ALA LYS	3.85E-09
molecular function	methyltransferase activity	GLU ALA LEU	4.58E-09
molecular function	pancreatic ribonuclease activity	PHE ASP VAL	8.81E-10
molecular function	pancreatic ribonuclease activity	ASP VAL ILE	5.14E-10
molecular function	pancreatic ribonuclease activity	PHE VAL ILE	7.40E-09
molecular function	pancreatic ribonuclease activity	ALA PHE LYS	3.61E-08
molecular function	pancreatic ribonuclease activity	VAL TYR ILE	3.40E-13
molecular function	kinase activity	GLU ALA ILE	5.89E-09
molecular function	kinase activity	GLU LEU ILE	1.50E-09
molecular function	kinase activity	GLU VAL LEU	1.10E-08
molecular function	metal ion binding	VAL LEU HIS	9.25E-09
molecular function	metal ion binding	ALA LEU HIS	5.79E-09
cellular component	mitochondrion	LYS TYR LEU	3.35E-08

cellular component	Golgi lumen	ALA TYR LEU	7.35E-08
cellular component	Golgi lumen	CYS VAL LEU	4.05E-10
cellular component	endosome lumen	ALA TYR LEU	7.35E-08
cellular component	endosome lumen	CYS VAL LEU	4.05E-10
cellular component	endoplasmic reticulum lumen	CYS VAL LEU	9.04E-08
cellular component	protein complex	GLU PHE ARG	1.03E-07
cellular component	protein complex	ASN VAL ILE	6.21E-08
cellular component	hemoglobin complex	CYS VAL LEU	1.10E-09
cellular component	hemoglobin complex	ALA LEU HIS	1.30E-10
cellular component	cytoplasm	LYS LEU GLN	2.71E-09
cellular component	cytoplasm	GLU LYS LEU	1.70E-08
cellular component	secretory granule	CYS VAL LEU	9.16E-10
cellular component	cytosol	PHE LEU SER	1.07E-08
cellular component	membrane	PHE VAL LEU	4.01E-08
cellular component	membrane	PHE THR VAL	5.44E-09
cellular component	membrane	PHE LEU SER	6.70E-09
cellular component	integral component of membrane	PHE LEU ILE	1.40E-14
cellular component	integral component of membrane	GLY TYR LEU	1.67E-09
cellular component	integral component of membrane	THR VAL LEU	3.49E-08
cellular component	integral component of membrane	PHE VAL LEU	1.72E-14
cellular component	integral component of membrane	PHE THR VAL	9.26E-13
cellular component	integral component of membrane	PHE VAL ILE	3.72E-09
cellular component	integral component of membrane	VAL LEU ILE	6.44E-08
cellular component	integral component of membrane	ALA PHE THR	4.44E-14
cellular component	integral component of membrane	ALA PHE ILE	4.27E-10
cellular component	integral component of membrane	TYR LEU SER	1.65E-11
cellular component	integral component of membrane	PHE LEU SER	4.24E-11
cellular component	nucleosome	ALA TYR LEU	3.03E-10
biological process	positive regulation of phosphatidylinositol 3-kinase signaling	CYS VAL LEU	1.49E-09
biological process	oxidation-reduction process	ALA LYS LEU	1.18E-13
biological process	oxidation-reduction process	GLU ALA VAL	3.06E-16
biological process	oxidation-reduction process	ALA LEU ARG	4.31E-12

biological process	oxidation-reduction process	ALA VAL ILE	4.57E-15
biological process	oxidation-reduction process	THR VAL LEU	1.44E-16
biological process	oxidation-reduction process	ALA THR LEU	3.06E-19
biological process	oxidation-reduction process	PHE VAL SER	1.72E-08
biological process	oxidation-reduction process	ASP VAL LEU	8.91E-10
biological process	oxidation-reduction process	GLU LEU ARG	1.38E-08
biological process	oxidation-reduction process	ALA LEU HIS	1.24E-11
biological process	oxidation-reduction process	PHE LEU SER	9.30E-08
biological process	oxidation-reduction process	ALA PHE ARG	5.95E-09
biological process	oxidation-reduction process	ALA PHE LEU	5.66E-17
biological process	oxidation-reduction process	ALA PHE SER	4.47E-12
biological process	oxidation-reduction process	GLU ALA ILE	6.75E-15
biological process	oxidation-reduction process	ALA THR VAL	6.30E-18
biological process	oxidation-reduction process	GLY TYR LEU	5.40E-10
biological process	oxidation-reduction process	ASN ALA ILE	1.11E-07
biological process	oxidation-reduction process	ALA ILE ARG	9.60E-08
biological process	oxidation-reduction process	ALA VAL LEU	3.49E-15
biological process	oxidation-reduction process	ALA PHE THR	1.34E-21
biological process	oxidation-reduction process	ALA PHE LYS	2.30E-14
biological process	oxidation-reduction process	ALA THR ILE	1.09E-12
biological process	oxidation-reduction process	ALA PHE VAL	1.01E-09
biological process	oxidation-reduction process	PHE LEU ARG	4.01E-08
biological process	oxidation-reduction process	VAL LEU HIS	3.83E-09
biological process	oxidation-reduction process	ALA LYS VAL	8.92E-08
biological process	oxidation-reduction process	VAL LEU ILE	3.69E-13
biological process	oxidation-reduction process	ASP LEU ARG	4.29E-09
biological process	oxidation-reduction process	ALA LEU GLN	4.88E-09
biological process	oxidation-reduction process	ALA VAL ARG	9.42E-14
biological process	oxidation-reduction process	GLU ALA ARG	2.21E-10
biological process	oxidation-reduction process	GLU ALA LEU	4.51E-21
biological process	oxidation-reduction process	ALA VAL TYR	7.61E-13
biological process	oxidation-reduction process	ALA LEU ILE	1.29E-16
biological process	oxidation-reduction process	ALA TYR LEU	2.76E-09
biological process	oxidation-reduction process	GLU ALA PHE	2.55E-21
biological process	oxidation-reduction process	ALA VAL GLN	2.23E-10
biological process	oxidation-reduction process	ALA ASP ILE	2.24E-10
biological process	negative regulation of gluconeogenesis	CYS VAL LEU	3.30E-10
biological process	positive regulation of protein autophosphorylation	CYS VAL LEU	4.05E-10
biological process	inflammatory response	CYS ALA LEU	3.65E-10
biological process	negative regulation of feeding behavior	CYS VAL LEU	9.22E-11
biological process	positive regulation of cellular protein metabolic process	CYS VAL LEU	8.58E-11

biological process	positive regulation of mitosis	CYS VAL LEU	9.22E-11
biological process	negative regulation of acute inflammatory response	CYS VAL LEU	1.37E-08
biological process	energy reserve metabolic process	CYS VAL LEU	2.51E-10
biological process	alpha-beta T cell activation	CYS VAL LEU	9.22E-11
biological process	fatty acid homeostasis	CYS VAL LEU	9.22E-11
biological process	negative regulation of protein secretion	CYS VAL LEU	9.22E-11
biological process	positive regulation of cell growth	CYS VAL LEU	1.27E-10
biological process	glucose metabolic process	CYS VAL LEU	1.12E-08
biological process	positive regulation of glucose import	CYS VAL LEU	1.87E-11
biological process	positive regulation of vasodilation	ALA TYR LEU	7.35E-08
biological process	positive regulation of vasodilation	CYS VAL LEU	1.87E-11
biological process	glucose transport	CYS VAL LEU	5.66E-11
biological process	regulation of protein secretion	CYS VAL LEU	3.58E-08
biological process	negative regulation of vasodilation	CYS VAL LEU	9.22E-11
biological process	defense response to bacterium	ALA VAL ARG	5.44E-08
biological process	defense response to bacterium	CYS ALA LEU	1.45E-19
biological process	positive regulation of DNA replication	CYS VAL LEU	1.37E-08
biological process	transport	PHE LYS LEU	1.51E-08
biological process	negative regulation of NAD(P)H oxidase activity	CYS VAL LEU	9.22E-11
biological process	protein heterooligomerization	PHE LEU SER	2.86E-08
biological process	negative regulation of fatty acid metabolic process	CYS VAL LEU	9.22E-11
biological process	positive regulation of peptide hormone secretion	CYS VAL LEU	9.22E-11
biological process	positive regulation of peptidyl-tyrosine phosphorylation	CYS VAL LEU	3.54E-10
biological process	small molecule metabolic process	CYS VAL LEU	3.92E-08
biological process	regulation of protein localization	CYS VAL LEU	3.16E-08
biological process	positive regulation of nitric-oxide synthase activity	CYS VAL LEU	1.87E-11
biological process	activation of protein kinase B activity	CYS VAL LEU	4.77E-09
biological process	regulation of transmembrane transporter activity	CYS VAL LEU	9.22E-11
biological process	protein acetylation	ASN VAL ILE	8.23E-08
biological process	protein acetylation	PHE LYS VAL	4.49E-08
biological process	protein acetylation	THR ILE ARG	8.87E-08
biological process	cilium or flagellum-dependent	GLU PHE LYS	6.24E-09

	cell motility		
biological process	cilium or flagellum-dependent cell motility	LYS LEU GLN	4.94E-08
biological process	cilium or flagellum-dependent cell motility	GLU PHE ILE	3.52E-10
biological process	cilium or flagellum-dependent cell motility	ASN VAL ILE	9.02E-11
biological process	positive regulation of nitric oxide biosynthetic process	CYS VAL LEU	3.74E-12
biological process	positive regulation of lipid biosynthetic process	CYS VAL LEU	1.87E-11
biological process	positive regulation of respiratory burst	CYS VAL LEU	9.22E-11
biological process	cell wall macromolecule catabolic process	CYS ALA LEU	1.74E-09
biological process	negative regulation of protein oligomerization	CYS VAL LEU	9.22E-11
biological process	insulin receptor signaling pathway	VAL TYR LEU	1.02E-08
biological process	retinol metabolic process	ASN VAL ILE	1.23E-09
biological process	retinol metabolic process	PHE LYS VAL	5.29E-10
biological process	negative regulation of lipid catabolic process	CYS VAL LEU	9.22E-11
biological process	flagellar cell motility	GLU PHE ILE	5.97E-08
biological process	flagellar cell motility	THR ILE ARG	2.29E-08
biological process	cellular amino acid biosynthetic process	ALA VAL ILE	7.56E-08
biological process	cellular amino acid biosynthetic process	ALA LYS LEU	5.25E-08
biological process	cellular amino acid biosynthetic process	GLU ALA ILE	8.87E-08
biological process	cellular amino acid biosynthetic process	GLU ALA VAL	1.06E-07
biological process	cellular amino acid biosynthetic process	ALA VAL LEU	1.82E-09
biological process	cellular amino acid biosynthetic process	GLU LEU ILE	6.03E-08
biological process	cellular amino acid biosynthetic process	GLU ALA LEU	3.44E-13
biological process	cellular amino acid biosynthetic process	ALA LEU ILE	2.80E-09
biological process	cytolysis	ASN ALA ILE	1.04E-08
biological process	cytolysis	ALA VAL ARG	1.61E-11
biological process	cytolysis	ALA ILE ARG	1.23E-08
biological process	cytolysis	CYS ALA LEU	1.72E-23
biological process	cytolysis	GLU ALA LYS	4.73E-08

biological process	negative regulation of glycogen catabolic process	CYS VAL LEU	9.22E-11
biological process	positive regulation of cytokine secretion	CYS VAL LEU	2.51E-10
biological process	negative regulation of protein catabolic process	CYS VAL LEU	1.87E-11
biological process	regulation of insulin secretion	CYS VAL LEU	8.58E-11
biological process	positive regulation of glycolytic process	CYS VAL LEU	4.05E-10
biological process	positive regulation of insulin receptor signaling pathway	CYS VAL LEU	9.22E-11
biological process	steroid metabolic process	PHE THR LEU	1.11E-07
biological process	wound healing	CYS VAL LEU	5.72E-09
biological process	positive regulation of glycogen biosynthetic process	CYS VAL LEU	8.58E-11
biological process	positive regulation of MAPK cascade	CYS VAL LEU	3.75E-12
biological process	phosphorylation	GLU ALA ILE	2.42E-08
biological process	phosphorylation	ALA THR ILE	1.12E-07
biological process	phosphorylation	GLU LEU ILE	5.51E-10
biological process	phosphorylation	GLU VAL LEU	4.86E-08
biological process	response to freezing	ASP ILE ARG	1.16E-07
biological process	glucose homeostasis	CYS VAL LEU	1.43E-09
biological process	regulation of cellular amino acid metabolic process	ALA TYR LEU	7.35E-08
biological process	regulation of cellular amino acid metabolic process	CYS VAL LEU	1.87E-11
biological process	growth	ALA VAL ARG	3.85E-10
biological process	growth	ASP LEU ARG	3.95E-09
biological process	positive regulation of cell differentiation	CYS VAL LEU	4.05E-10
biological process	methylation	GLU LEU ARG	3.20E-08
biological process	methylation	GLU ALA LEU	8.74E-10
biological process	metabolic process	ALA LYS LEU	7.60E-08
biological process	metabolic process	CYS ALA LEU	6.17E-08
biological process	metabolic process	ALA LEU ARG	1.63E-12
biological process	metabolic process	ASN ALA LEU	1.52E-09
biological process	metabolic process	ALA THR LEU	1.89E-12
biological process	metabolic process	GLU LEU ARG	5.04E-17
biological process	metabolic process	ALA PHE ILE	1.01E-08
biological process	metabolic process	ALA THR VAL	1.67E-09
biological process	metabolic process	ALA VAL ARG	2.88E-26
biological process	metabolic process	GLU ALA ARG	4.06E-11
biological process	metabolic process	GLU ALA LYS	3.41E-13
biological process	metabolic process	GLU ALA LEU	2.60E-20

biological process	metabolic process	ALA VAL TYR	1.34E-08
biological process	metabolic process	ALA LEU ILE	4.43E-08
biological process	metabolic process	ASP VAL ILE	8.57E-08
biological process	metabolic process	ALA ASP ILE	6.95E-16
biological process	metabolic process	ALA ILE ARG	7.02E-14
biological process	positive regulation of brown fat cell differentiation	CYS VAL LEU	9.22E-11
biological process	negative regulation of proteolysis	CYS VAL LEU	1.87E-11
biological process	endocrine pancreas development	CYS VAL LEU	8.58E-11
biological process	carbohydrate metabolic process	GLU ALA ILE	1.15E-07
biological process	carbohydrate metabolic process	GLU ALA VAL	4.79E-09
biological process	carbohydrate metabolic process	GLU ALA LEU	6.50E-11
biological process	negative regulation of respiratory burst involved in inflammatory response	CYS VAL LEU	9.22E-11
biological process	oxygen transport	ALA THR VAL	2.50E-09
biological process	oxygen transport	ALA LEU HIS	1.46E-10
biological process	oxygen transport	ALA PHE LYS	5.23E-09
biological process	oxygen transport	PHE LYS LEU	1.75E-16
biological process	oxygen transport	PHE LYS VAL	3.14E-10
biological process	acute-phase response	CYS VAL LEU	7.22E-11
