# Supplementary material for: Mining the entire Protein DataBank for frequent spatially cohesive amino acid patterns
Source: BioData Min. 2015 Jan 31;8:4. doi: 10.1186/s13040-015-0038-4 (PMC4318390; doi:10.1186/s13040-015-0038-4)

Additional file 9: Distribution of the distance along the protein sequence between residues captured for each FreSCO match across all analysed protein structures. Distances were transformed with natural logarithm for visualisation purposes. Residues within a distance of 5 (~1.6 in the graph) can be considered as 'short-range' contacts. FreSCOs spanning more than 10 residues (~2.3 in the graph) are defined as including 'long-range' contacts.

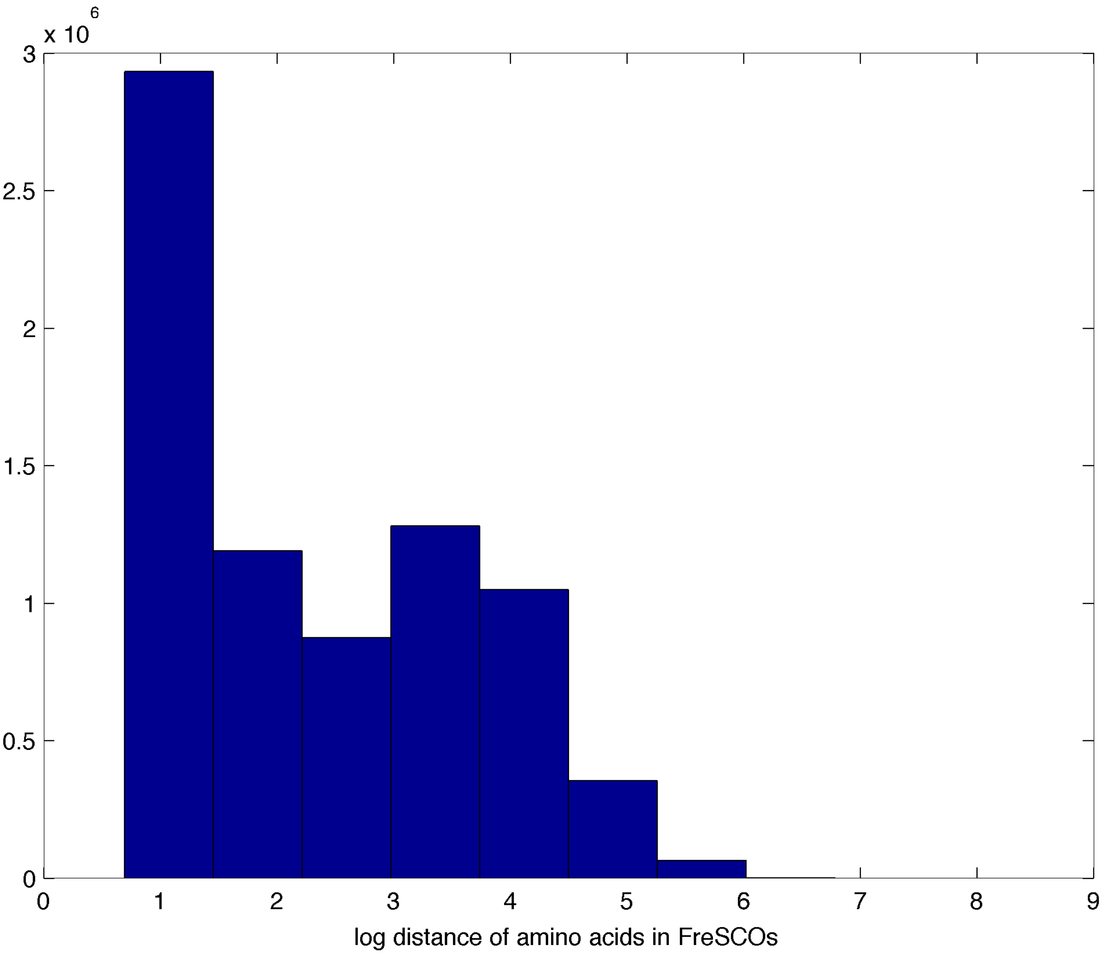

Supplement: Additional file 7: — Distribution of the distance along the protein sequence between residues captured for each FreSCO match. [file 13040_2015_38_MOESM7_ESM.pdf]
