# Supplementary material for: Mining the entire Protein DataBank for frequent spatially cohesive amino acid patterns
Source: BioData Min. 2015 Jan 31;8:4. doi: 10.1186/s13040-015-0038-4 (PMC4318390; doi:10.1186/s13040-015-0038-4)
Supplement: Additional file 8: — Mined FreSCOs significantly correlated with optimal growth temperature. [file 13040_2015_38_MOESM8_ESM.pdf]

Additional file 5: Mined amino acid FreSCOs significantly correlated with optimal growth temperature.

Mined FreSCOs found to be significant correlated with high OGT. Significance cut-off was 3.84E-5.

FreSCO	P-value
GLU LYS VAL	1.34E-100
GLU LYS LEU	2.30E-100
GLU LYS ILE	4.37E-90
GLU LYS ARG	3.74E-82
LYS VAL ARG	1.09E-73
LYS ILE ARG	2.12E-66
LYS LEU ARG	3.03E-66
LYS VAL ILE	7.00E-64
LYS VAL LEU	1.00E-61
GLU GLY LYS	2.21E-55
GLU ALA LYS	1.56E-49
GLU PHE LYS	1.11E-47
LYS LEU ILE	3.44E-47
GLY LYS ARG	6.28E-46
GLU LYS ASP	4.64E-42
GLU VAL ARG	5.77E-42
LYS VAL TYR	1.93E-39
LYS TYR LEU	6.34E-39
PHE LYS LEU	8.58E-39
GLU ILE ARG	8.43E-37
GLY LYS LEU	1.54E-36
GLU LEU ARG	1.75E-36
GLU LYS SER	1.89E-35
ALA LYS ARG	2.01E-34
GLU VAL ILE	5.52E-34
GLY LYS VAL	2.44E-32
GLY LYS ILE	1.97E-31
LEU ILE ARG	4.25E-30
LYS PRO LEU	7.53E-28
GLU LEU ILE	1.20E-26
GLU VAL LEU	2.76E-26
GLU THR LYS	7.82E-25
LYS SER ILE	2.49E-24
ALA LYS VAL	2.75E-24
ALA LYS ILE	5.02E-24
VAL ILE ARG	2.43E-23
LYS ASP VAL	2.61E-23
ALA LYS LEU	2.04E-22

PHE LYS VAL	8.06E-22
GLU TYR LEU	1.52E-21
GLU GLY ILE	1.27E-20
GLU PHE ILE	2.95E-19
VAL LEU ILE	7.25E-19
GLU PHE ARG	1.25E-18
LYS ASP LEU	1.34E-18
GLU GLY ARG	1.49E-18
LYS VAL SER	1.89E-18
GLU GLY VAL	1.38E-17
GLU PHE LEU	1.42E-17
GLU VAL TYR	1.69E-17
ASN LYS LEU	3.11E-17
GLU PHE VAL	4.96E-17
GLU GLY LEU	2.00E-16
GLU PRO VAL	1.24E-15
GLU ASP VAL	3.98E-15
LYS ASP ILE	4.08E-14
VAL TYR ILE	5.69E-14
GLU PRO LEU	1.00E-13
VAL LEU ARG	7.97E-13
THR LYS ILE	2.24E-12
GLU ALA ARG	7.21E-12
PRO LEU ILE	1.37E-11
VAL TYR LEU	2.20E-11
GLU ALA ILE	1.38E-10
ASN GLU LEU	1.44E-10
PRO VAL ILE	2.95E-10
GLU ASP LEU	7.04E-10
LYS LEU SER	1.01E-09
GLY LYS ASP	1.20E-09
GLY ILE ARG	1.30E-09
TYR LEU ILE	1.62E-09
PHE LEU ILE	2.07E-09
GLY THR LYS	5.85E-09
ASN GLU VAL	9.85E-09
GLU ASP ARG	2.32E-08
ALA ILE ARG	4.37E-08
GLU ASP ILE	9.22E-08
PRO VAL LEU	1.01E-07
ALA GLY LYS	1.15E-07
GLU ALA LEU	1.29E-07
PHE VAL ILE	1.35E-07
GLY LYS SER	1.39E-07
PHE VAL ARG	2.31E-07

GLU PHE GLY	5.56E-07
PHE LEU ARG	8.93E-07
ALA PHE LYS	8.97E-07
GLU ALA VAL	9.13E-07
GLY VAL ARG	1.25E-06
THR LYS LEU	1.62E-06
GLY VAL ILE	2.44E-06
TYR LEU ARG	3.00E-06
ASN LEU ARG	1.13E-05
GLU SER ILE	1.26E-05
GLY LEU ILE	1.32E-05
GLU SER ARG	2.17E-05

Mined FreSCOs found to be significant correlated with low OGT. Significance cut-off was 3.84E-5.

FreSCO	P-value
ALA THR ASP	3.27E-55
ALA ASP SER	2.64E-45
THR LEU GLN	5.69E-45
ASP LEU GLN	2.57E-40
ALA GLY ASP	9.03E-36
ALA GLY GLN	1.37E-34
LEU GLN SER	3.94E-32
GLY LEU GLN	6.54E-30
ALA LEU GLN	2.38E-26
ALA VAL GLN	4.38E-25
ALA THR SER	4.40E-25
GLY THR ASP	2.11E-23
ALA PHE THR	1.66E-21
GLY VAL GLN	1.79E-19
ALA PHE SER	1.76E-18
GLY ASP SER	1.05E-17
VAL LEU GLN	5.59E-17
ALA THR LEU	6.48E-17
ALA GLY SER	6.89E-17
ALA PHE ASP	1.86E-16
ALA GLN ILE	5.72E-16
LEU GLN ARG	1.23E-15
GLU ALA THR	2.56E-14
ALA ASP LEU	4.12E-14
ALA GLY THR	7.89E-14
GLU ALA GLN	1.63E-13
THR ASP LEU	3.35E-13

CYS ALA LEU	5.11E-13
THR ASP ILE	1.60E-11
GLY THR SER	1.97E-11
ALA THR VAL	4.89E-11
GLY THR LEU	1.15E-10
ASP LEU SER	2.12E-10
THR ASP VAL	3.47E-10
THR LEU SER	4.16E-10
ALA ASP ILE	2.74E-09
VAL GLN ILE	9.34E-09
GLU THR SER	2.63E-08
ASN THR LEU	2.86E-08
PHE THR VAL	3.27E-08
ALA THR ARG	3.51E-08
PHE THR LEU	5.03E-08
ASN LEU SER	2.03E-07
LEU GLN ILE	2.43E-07
ALA ASP VAL	3.35E-07
GLU LEU GLN	6.17E-06
ALA GLY TYR	7.48E-06
ALA LEU SER	1.13E-05
ALA ASP ARG	2.64E-05
