# Supplementary material for: Mining the entire Protein DataBank for frequent spatially cohesive amino acid patterns
Source: BioData Min. 2015 Jan 31;8:4. doi: 10.1186/s13040-015-0038-4 (PMC4318390; doi:10.1186/s13040-015-0038-4)
Supplement: Additional file 9: — Correlation of individual amino acids in a protein and the associated OGT. [file 13040_2015_38_MOESM9_ESM.pdf]

Additional file 6: Spearman correlation of occurrence of individual amino acids in a protein and the associated OGT. Negative correlation means the amino acid is more frequent in proteins associated with a low OGT, positive correlation with high OGT.

Amino acid	Correlation	P-value
GLN	-0.147	6.51E-104
ASP	-0.123	1.08E-73
THR	-0.113	1.56E-61
HIS	-0.105	1.33E-53
ALA	-0.0861	1.29E-36
SER	-0.0685	8.98E-24
ASN	-0.0537	3.60E-15
TRP	-0.0356	1.89E-07
PHE	-0.0171	1.22E-02
CYS	-0.00364	0.594
UNK	-0.00246	0.718
PRO	-0.00165	0.808
MET	0.0126	0.0702
LEU	0.0274	5.93E-05
TYR	0.0293	1.78E-05
ILE	0.0296	1.44E-05
GLY	0.0399	5.04E-09
ARG	0.0719	5.61E-26
VAL	0.0895	2.08E-39
LYS	0.104	2.30E-52
GLU	0.127	1.94E-78
