# Supplementary material for: Mining the entire Protein DataBank for frequent spatially cohesive amino acid patterns
Source: BioData Min. 2015 Jan 31;8:4. doi: 10.1186/s13040-015-0038-4 (PMC4318390; doi:10.1186/s13040-015-0038-4)
Supplement: Additional file 10: — Overview of the significance of the found amino acid–base FreSCOs in the protein-DNA complex structure data set. [file 13040_2015_38_MOESM10_ESM.pdf]

Additional file 7: Overview of the significance of the found amino acid-base FreSCOs in the protein-DNA complex structure dataset. Frequency and cohesion radius of the significant amino acid-base patterns. P-value based on the cohesion radius of the same FreSCOs in permutated DNA-protein structures.

FreSCO	Frequency	Cohesion radius	P-value
DT GLY ARG	0.939676	6.127467	2.97E-283
DT GLY	0.939676	4.530339	4.51E-266
DT GLY SER	0.935195	6.155224	1.95E-209
DT THR GLY	0.935195	6.386277	2.00E-158
DT GLY ALA	0.936574	6.389396	1.46E-108
DT SER	0.943123	4.683291	9.63E-100
DA DT DG SER	0.89314	6.752896	4.90E-97
DT LYS ASN	0.940021	6.627604	1.00E-92
DC DA DG SER	0.892106	6.796654	3.98E-86
DT LYS SER	0.942778	6.13308	8.37E-82
DA DT DG ARG	0.896932	6.617025	4.68E-79
DT ARG SER	0.943123	6.229689	2.21E-78
DC GLY ARG	0.922096	6.308396	7.48E-78
DC GLY	0.922096	4.890112	9.94E-75
DT THR SER	0.939676	6.496252	1.35E-71
DT SER ASN	0.937608	6.809997	2.21E-61
DT LYS THR	0.942778	6.349891	4.12E-58
DA DT DG GLY	0.889004	6.7881	1.08E-57
DC THR GLY	0.916925	6.672068	6.23E-54
DC DT GLY	0.895209	6.047739	9.24E-54
DC LYS GLY	0.921751	6.251711	2.82E-53
DC LYS THR	0.924509	6.524064	2.54E-51
DA DT GLY	0.896932	6.10275	4.48E-50
DT LYS GLY	0.939331	6.169234	3.84E-49
DT ASN	0.940021	5.325566	1.60E-48
DT GLY PRO	0.927956	6.920673	3.68E-47
DT LYS ARG	0.94726	6.160239	8.12E-46
DC LYS ARG	0.929679	6.166498	5.97E-43
DC THR ARG	0.924853	6.571643	1.42E-41
DT GLY ASN	0.932092	6.922134	8.47E-40
DT GLN SER	0.932092	6.923004	3.25E-39
DC SER	0.924509	4.8843	5.42E-38
DC GLY SER	0.91658	6.414189	6.40E-37
DC DA DG GLY	0.889004	6.89531	1.27E-36
DT ILE GLY	0.930024	6.582475	1.31E-36
DA DT ASN	0.897277	6.770477	2.05E-35
DA DT DG	0.905895	2.950319	3.32E-35
DC LYS SER	0.924164	6.348909	6.06E-35
DA DG SER	0.903137	6.234865	7.44E-35

DC DA DG ARG	0.896932	6.716098	1.80E-34
DT THR ARG	0.943123	6.482018	1.86E-33
DC ARG ASN	0.921751	6.755637	4.72E-33
DA DG ARG	0.907963	6.089308	1.35E-32
DT ARG ASN	0.940021	6.777521	8.04E-32
DC ARG SER	0.924509	6.374127	8.82E-32
DC THR SER	0.921062	6.683413	5.35E-31
DT THR ASN	0.936574	6.987177	6.83E-31
DA DT SER	0.901413	6.211182	2.70E-30
DC DA DT GLY	0.882799	6.885953	1.73E-29
DT ILE ASN	0.932437	6.976099	2.03E-29
DA DT ARG	0.905205	6.074988	5.85E-28
DT THR	0.947949	5.079601	8.54E-28
DC DT DG SER	0.890038	6.941497	1.41E-24
DT GLY VAL	0.931058	6.621836	5.37E-24
DC LYS ASN	0.921751	6.834252	9.23E-24
DT ILE SER	0.935884	6.674606	2.59E-22
DC DT DG GLY	0.886936	6.9326	1.28E-21
DT ILE THR	0.934161	6.804892	6.87E-20
DA DG GLY	0.900034	6.276371	1.78E-19
DC DG SER	0.906929	6.245179	2.35E-19
DC DG GLY	0.904516	6.2695	3.78E-19
DC DT SER	0.898311	6.175282	4.97E-19
DC DA DG LYS	0.896932	6.751376	1.31E-18
DC DA DT SER	0.886936	6.945109	1.34E-18
DC DT LYS	0.903137	6.000176	3.24E-18
DC DA DT ARG	0.890727	6.794205	1.55E-17
DC ASN	0.921751	5.436475	2.21E-17
DC DT ASN	0.895209	6.70148	2.88E-17
DT ARG	0.947949	4.800107	5.10E-17
DC ALA SER	0.923819	6.537923	6.55E-16
DA DT DG LYS	0.896932	6.803256	1.21E-15
DT ALA SER	0.941744	6.558472	8.41E-15
DA DG ASN	0.89969	6.87482	1.37E-14
DC DT ARG	0.903137	6.04922	6.88E-14
DC ARG	0.930369	4.857176	3.00E-13
DT LYS	0.947949	4.771483	1.69E-12
DA DT THR	0.905205	6.563358	2.11E-11
DC DT DG ARG	0.894864	6.807256	6.53E-11
DT THR ALA	0.941055	6.664431	2.02E-10
DT DG GLY	0.90555	6.277393	1.32E-09
DT SER VAL	0.935884	6.714867	2.04E-09
DC DG ASN	0.904516	6.776123	3.55E-09
DA GLY ARG	0.918649	6.703545	6.01E-09
DC DA DT LYS	0.890727	6.8367	1.32E-08

DT DG ARG	0.913478	6.159504	1.71E-08
DT DG SER	0.908652	6.307371	4.62E-08
DT THR VAL	0.935195	6.820829	1.95E-07
DA DT LYS	0.905205	6.185748	1.12E-06
DC ILE GLY	0.911755	6.794858	1.49E-06
DC GLY ALA	0.919683	6.691878	1.54E-06
DC DT DG LYS	0.894864	6.827585	1.73E-06
DC DT THR	0.902792	6.529231	3.52E-06
DT ILE ARG	0.937608	6.724475	3.98E-06
DC DG ARG	0.912789	6.13217	7.76E-06
